# Promoting gender equality across the sustainable development goals

**DOI:** 10.1007/s10668-022-02656-1

**Published:** 2022-09-15

**Authors:** Walter Leal Filho, Marina Kovaleva, Stella Tsani, Diana-Mihaela Țîrcă, Chris Shiel, Maria Alzira Pimenta Dinis, Melanie Nicolau, Mihaela Sima, Barbara Fritzen, Amanda Lange Salvia, Aprajita Minhas, Valerija Kozlova, Federica Doni, Jane Spiteri, Tanushka Gupta, Kutoma Wakunuma, Mohit Sharma, Jelena Barbir, Kalterina Shulla, Medani P. Bhandari, Shiv Tripathi

**Affiliations:** 1 Research and Transfer Centre “Sustainable Development and Climate Change Management”, Hamburg University of Applied Sciences, Ulmenliet 20, 21033 Hamburg, Germany; 2 School of Science and the Environment, Manchester Metropolitan University, All Saints Building, Oxford Road, Manchester, M15 6BH UK; 3 Department of Economics, University of Ioannina, University Campus, 451 10 Ioannina, Greece; 4 Faculty of Economics, Department of Management and Business Administration, “Constantin Brâncuși” University of Târgu-Jiu, Str. Tineretului, Nr. 4, Târgu-Jiu Gorj, Romania; 5 Department of Life & Environmental Science, Bournemouth University, Poole Dorset, BH12 5BB UK; 6 UFP Energy, Environment and Health Research Unit (FP-ENAS), University Fernando Pessoa (UFP), Praça 9 de Abril 349, 4249-004 Porto, Portugal; 7 Department of Geography, University of South Africa, Private Bag X6, Florida, 1710 South Africa; 8 Environment and GIS Department, Institute of Geography, Romanian Academy, 12 Dimitrie Racovita St., Sector 2, 023993 Bucharest, Romania; 9 University of Passo Fundo, BR 285, São José, Passo Fundo, Rio Grande do Sul 99052-900 Brazil; 10 Graduate Program in Civil and Environmental Engineering, University of Passo Fundo, BR 285, São José, Passo Fundo, Rio Grande do Sul 99052-900 Brazil; 11 Faculty of Business and Economics, RISEBA University of Applied Sciences, Meza iela 3, Riga, 1048 Latvia; 12 Department of Business and Law, University of Milano-Bicocca, Via Bicocca degli Arcimboldi, 8, 20126 Milan, Italy; 13 Department of Early Childhood and Primary Education, Faculty of Education, University of Malta, Room 234, Old Humanities Building, Msida, MSD 2080 Malta; 14 Great Lakes Institute of Management, Chennai, Tamil Nadu 600041 India; 15 Centre for Computing and Social Responsibility, De Montfort University, Gateway House, Leicestershire, LE1 9BH UK; 16 Department of Public Policy and Public Administration, Central University of Jammu, Rahya Suchani, District- Samba, Bagla, J&K 181143 India; 17 Akamai University, 3211, Gibson Road, Durham, NC 27703 USA; 18 Sumy State University, Petropavlivska str, 57, Educational building К2, Cabinets 347-361, Sumy, 40000 Ukraine; 19 Institute of Health Management and Research, IIHMR University, 1, Prabhu Dayal Marg, Jaipur, 302029 India

**Keywords:** Sustainable development goals (SDGs), Gender equality, SDG5, Gender across SDGs

## Abstract

Gender issues, and gender equality in particular, can be regarded as cross-cutting issues in the implementation of the Sustainable Development Goals (SDGs), even though it is unclear how they are taken into account. This study addresses this information gap by performing an assessment of the emphasis on gender issues across all the other 16 SDGs, in addition to SDG5, through a literature review and case study analysis, the basis for the newly developed framework, highlighting specific actions associated to each SDG. The 13 countries addressed in the 16 case studies include China, India, or Australia and illustrate the inclusion of SDG5 into the SDGs. Using an SDG matrix, the SDG targets are analysed. Those where an emphasis on gender equality is important in allowing them to be achieved are listed. The novelty of our approach resides in offering an in-depth analysis of how gender issues interact with the other SDGs, proposing a new analysis framework clearly identifying SDGs 1, 4, 11, 12, 14 and 16 demanding further attention for successful SD gender implementation and illustrating specific areas where further actions may be necessary, which may be used by policy-makers, raising further awareness on gender equality contribution to achieve the SDGs. A set of recommendations aimed at placing gender matters more centrally in the SDGs delivery are presented as a final contribution. These focus on the need for greater awareness and attention to good practices, to achieve successful implementation initiatives.

**Supplementary Information:**

The online version contains supplementary material available at 10.1007/s10668-022-02656-1.

## Introducing SDG5–gender equality

In an unprecedented global effort, the heads of state and government and high representatives in the United Nations (UN) meeting of September 2015 put forward the ‘2030 Agenda’, a global plan for human and environmental prosperity, structured in 17 Sustainable Development Goals (SDGs) and 169 targets, indicative of the scale and of the ambition of the global action to be pursued. The 2030 Agenda recognises that the achievements of the 17 SDGs are linked to human and planetary prosperity, strengthening universal peace, greater freedom and promoting the eradication of poverty, discrimination and inequalities in all forms (UN, 2015). In the collective journey of meeting the SDGs and the UN 2030 Agenda targets, countries and stakeholders will act in partnership (Leal Filho et al., 2022a) to take a transformative and inclusive path towards a resilient and sustainable future in economic, social and environmental terms. The 2030 Agenda plans for the SDGs and the related targets trigger action in critical areas for human and planetary welfare. These include (UN, 2015): (i) human existence in prosperity, equality and a healthy environment, (ii) planet conservation through timely climate action, sustainable production, consumption and management of natural resources, (iii) economic, social and technological prosperity in a harmonious symbiosis with nature, (iv) peaceful, just and inclusive societies and (v) revived global partnership of countries, stakeholders and people.

SDG5, ‘Achieve gender equality and empower all women and girls’, reflects the ever-increasing efforts of the UN towards gender equality, earmarked with the establishment of the Commission on the Status of Women in 1946 (UN Women, 2020a) and the adoption of landmark agreements such as the Convention on the Elimination of All Forms of Discrimination against Women in 1979 (OHCHR, 2020), the Beijing Declaration and Platform for Action in 1995 (UN, 1995), and the establishment of UN Women in 2010 (UN, 2012). The important role of gender equality for socio-economic development is well highlighted in the UN publication “We the Peoples” (Annan, 2000), emphasising the untapped development potential due to social, economic and political inequalities arising from gender discrimination, deeply rooted and persistent in many developing and developed economies, related to access to decent work and equal pay, education, healthcare, resources, decision-making, among others (Brixiová et al., 2020; Tsige et al., 2020; Connor et al., 2020; Maheshwari & Nayak, 2020). Women are still more vulnerable to violence, discrimination, and underrepresentation in the political, economic, and business spheres (Milazzo & Goldstein, 2019; European Commission, 2019). The recognition of the important role of women in global, social, economic and environmental prosperity is clearly stated in paragraphs 236–243 of the ‘Future We Want’ (UN, 2012) and in the Open Working Group Proposal for Sustainable Development Goals (2014).

SDG5 brings forward issues of gender-based discrimination such as unpaid work, sexual and reproductive rights, and gender-based violence (Hirsu et al., 2019). Achieving SDG5 is a priority that contributes to the increase in global well-being. SDG5 includes nine targets that aim at ending all forms of discrimination, as described in Table 1. These targets set the sustainable development (SD) goals to be achieved. The indicators provide the monitoring approaches for status, progress, and assessment, chosen according to the respective objectives and measured globally, or at regional and country levels.

**Table 1:** SDG5 Targets and Indicators from UN (2021)

Target	Indicators
5.1 End discrimination against women and girls	5.1.1 Legal frameworks for gender equality and non-discrimination
5.2 End all violence against and exploitation of women and girls	5.2.1 Violence against women from an intimate partner
5.3 Eliminate forced marriages and genital mutilation	5.3.1 Women married before age 15 or 18 5.3.2 Female genital mutilation/cutting
5.4 Value unpaid care and promote shared domestic responsibilities	5.4.1 Time spent on unpaid domestic and care work
5.5 Ensure full participation in leadership and decision-making	5.5.1 Women in political positions 5.5.2 Women in managerial positions
5.6 Universal access to reproductive rights and health	5.6.1 Women’s decision-making on contraceptive use and healthcare 5.6.2 Guarantee of equal access to sexual and reproductive health care
5.A Equal rights to economic resources, property ownership, and financial services	5.A.1 Female land rights or ownership 5.A.2 Equal rights to land ownership
5.B Promote empowerment of women through technology	5.B.1 Mobile telephone ownership
5.C Adopt and strengthen policies and enforceable legislation for gender equality	5.C.1 Systems to track gender equality

But despite the relevance of the SDGs as a whole and the importance of handling gender issues, in particular, there is a research gap when it comes to looking at both topics in a combined way. In order to address this research need, this paper reports on a study aimed at fostering a thorough assessment of the emphasis that gender issues should be given in order to achieve all the SDGs. The research question pursued by the paper is the following: *o what extent are gender issues being considered in the overall implementation of the SDGs?*

Through a literature analysis and 16 case studies discussion in a sample of 13 developed and developing countries, e.g., China, India, Spain, and Morocco, this study sheds some light on the topic. The novelty behind this study consists in not only offering a sound analysis of how gender is considered across all other SDGs, but also indicating areas where further actions may be required. The innovation of this work is also based on the fact that it offers specific insights into gender equality and the SDGs. Also, this study may offer further guidance to policy-makers, thus prioritising women’s empowerment in developing collaborative initiatives in the area of gender equality. Finally, this paper also serves the purpose of raising awareness about the need for capacity building and sensitisation around gender-related issues and their crucial contribution to the SDGs.

## Research on gender equality and the SDGs: assessing the relations

SDGs have clear, often measurable and very straightforward targets aiming to improve the quality of life and living conditions for all. The interactions between these goals and the larger policy frameworks aiming to ensure economic growth from the country level to the regional level turns out to be more complex and challenging due to numerous types of constraints, from financial to cultural, when considering gender equality and ways of promoting it.

The global agenda for change, intent, purpose and overall goals were generally defined with the publishing of the Brundtland Report (1987), and the progress since then entered a new phase when the SDGs were adopted by the UN as the 2030 Agenda, while SD has been adopted across several economic policy fields in order to define specific objectives and goals. While highlighting SD challenges and opportunities, studies have included the gender dimension to a lesser extent, as illustrated in the existing literature that concerns the SDGs (Magendane & Kapazoglou, 2021; Scharlemann et al., 2020).

Gender inequality is pervasive across the world and women experience a series of disadvantages, in comparison to men. Yet, SD requires that we should all enjoy equal rights and be able to appreciate lives, free from violence and discrimination (UN Women, 2020a). There has been progress in some areas of discrimination, e.g., more girls in education, fewer girls forced into marriage, and more women in leadership roles, but policy decisions related to education, health and other sectors continue to take place in gendered contexts (Morgan et al., 2020). A situation where approximately half of the population is denied equal opportunities, equal participation in decision-making, and equal access to resources, education and employment will contribute to severely inhibiting SD and global prosperity (Dugarova, 2018).

Thus, and through SDG5, gender equality is rightfully at the heart of the 2030 Agenda for SD (UN, 2015), recognised as an essential human right and important enough to be a goal in its own right, among other 16 SDGs*.* Its significance is such that it constitutes a cross-cutting theme spanning all the other 16 SDGs, with a total of 45 targets and 54 indicators gender-related. It is suggested that not only is SDG5 critical to all the other SDGs, with gender inequality being an obstacle to progress, but that it has the potential to serve as an SD accelerator, with a positive multiplier effect, to speed up the progress of the 2030 Agenda (UNSDG, 2018). Gender equality and women’s empowerment should have a catalytic effect on human development (Odera & Mulusa, 2020) if gender is in fact actively addressed across all SDGs.

**Fig. 1:**
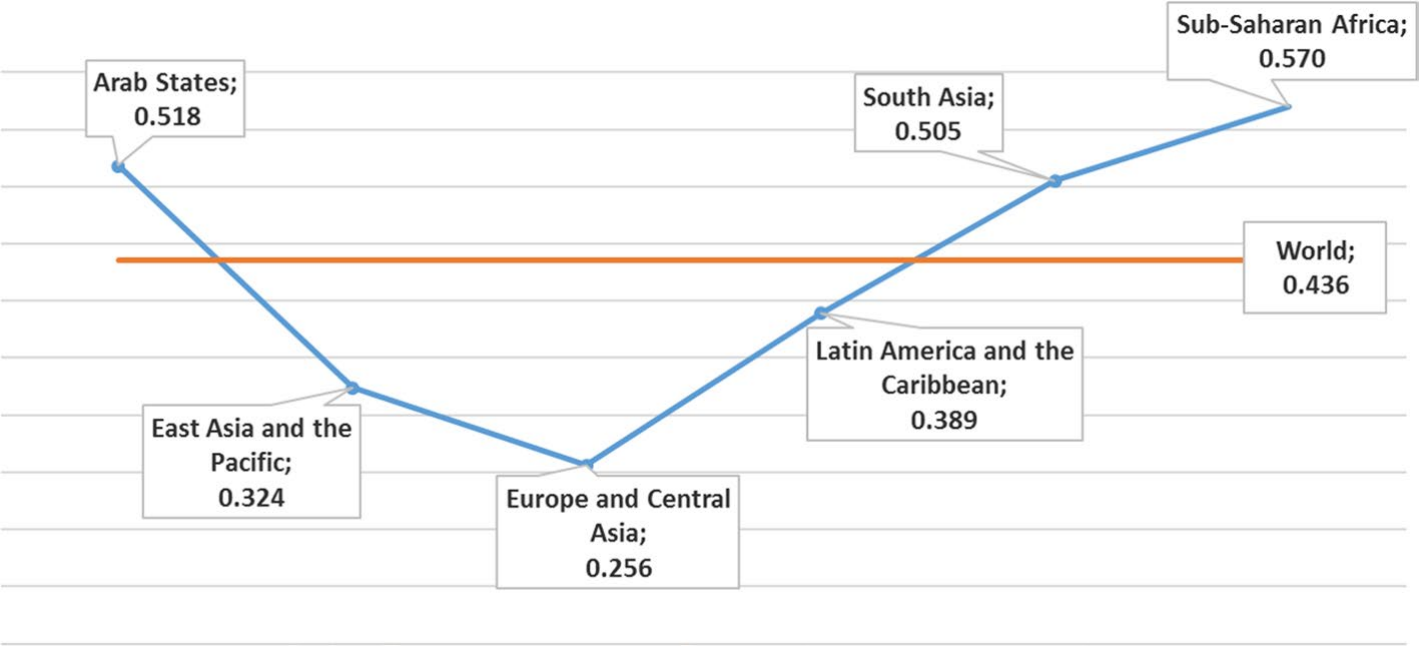
Gender Inequality Index, by developing region, 2020, modified from UNDP (2020)

**Fig. 2:**
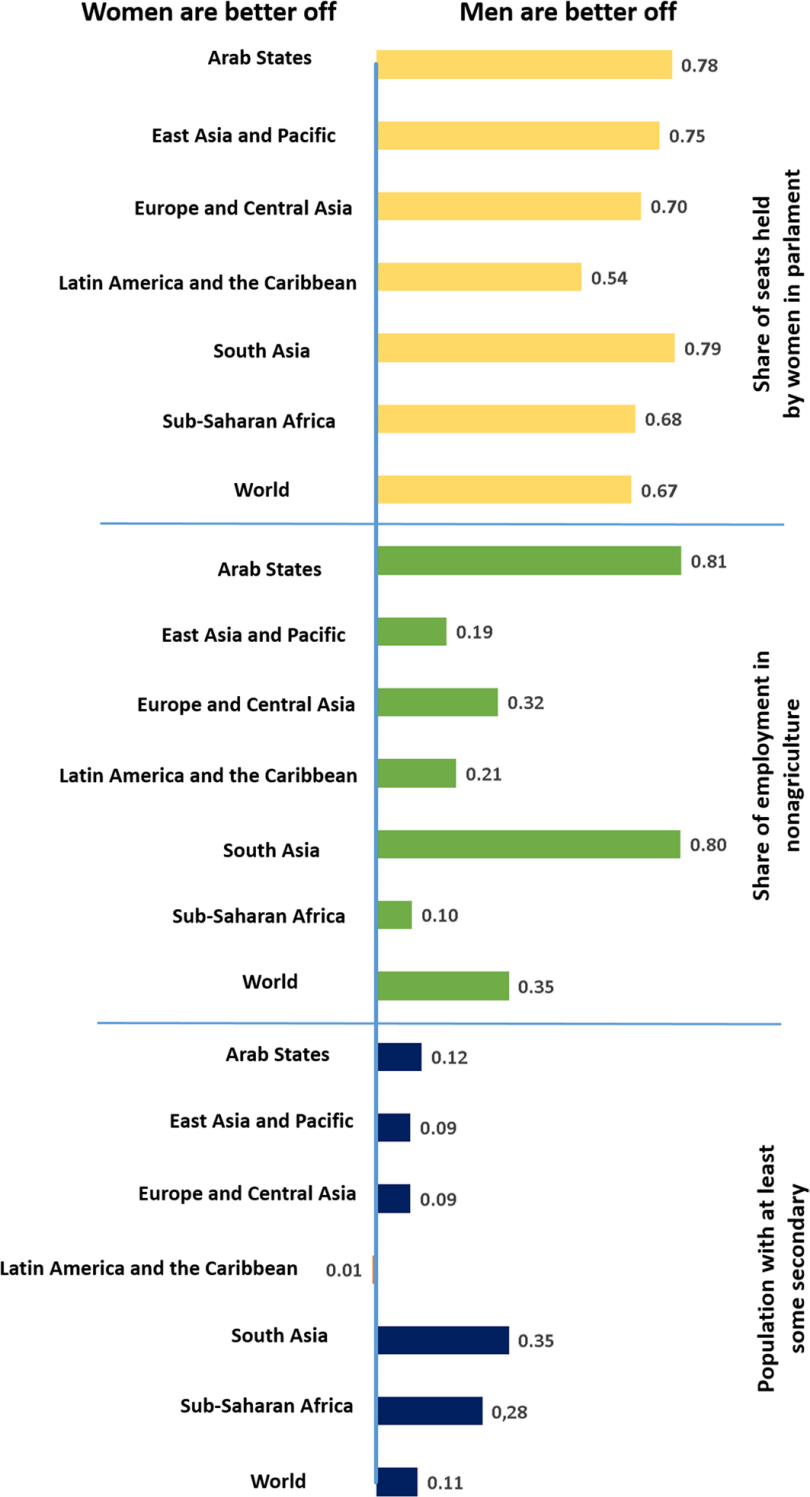
Life-course Gender Gap, 2019, modified from UNDP (2020)

There are a number of reasons why gender equality has to be considered in relation to all of the SDGs. If under-utilising part of the world’s talent, we fall short of reducing poverty (SDG1) and encouraging economic growth (SDG8). Gender equality in education and the labour market contributes to enhancing the gross domestic product and should help to reduce extreme poverty by 2030 (Dugarova, 2018). Compared to 1998, the gender gaps in the labour force, measured as the difference between the labour force participation rates of women and men, have decreased in most regions of the world in 2018, particularly in Latin America and the Caribbean, and Northern, Southern and Western Europe, but the gap has widened in Eastern Asia and Eastern Europe (Klasen, 2018). According to world regions, the Gender Inequality Index of 2020, can be seen in Fig. 1. Also, the Life-course Gender Gap in 2019, translating into a deviation from gender parity, reveals the gaps in the adult population (Fig. 2). The studies on gender equality reveal that women worldwide are more fragile in aspects such as poverty, representativeness in public employment positions, insecurity, or physical and sexual violence, thus emphasising the need to ensure a redesigned gender-responsive approach towards implementing the 2030 Agenda (Hirsu et al., 2019; Liu et al., 2019; Bourgault et al., 2021). Dugarova (2018) also demonstrates the multiple benefits of gender equality in relation to SD beyond SDG5, including food security, agricultural production, climate change (Caridade et al., 2022) and natural resource management. Similarly, Morgan et al. (2020) raise similar points but focusing on showing the importance of gender in relation to health and well-being (SDG3) and the less obvious connection between water and sanitation (SDG6) and energy (SDG7), illustrating the interconnected nature of SD and meaning that gender equality plays, in fact, an integral role to achieving all of the SDGs. Women are more likely to be impacted by unsafe water and poor sanitation (SDG6) and to die from unclean fuel (SDG7) (James et al., 2020), than men.

The World Employment and Social Outlook suggests that women are underpaid and under-employed (ILO, 2018), although playing a central role in the household economy and being important influencers in peaceful societies. The study from Manandhar et al. (2018) suggests that the concept of gender in SDG5, seeking the achievement of gender equality and empowerment of all women and girls, is narrow, focusing on women-specific limited roles. When considered in terms of social context impact, gender inequality affects justice in opportunities, leading to economic inefficiency and thus inhibiting growth and global SD (de Jong & Vijge, 2021).

According to Agarwal (2018), a bold interpretation of SDG5 and the establishment of synergies with the other SDGs could allow ways for women to contribute to progress in different aspects concerning SD. Asadikia et al. (2020) show the lack of influence that SDG5 alone has on an SDG index based on all observations, clearly highlighting the need to interact with other SDGs to increase SDG5 influence. Accordingly, it is important that other SDGs should refocus on the interactions of gender equality to achieve specific global sustainability objectives by 2030. Fariña García et al. (2020) used a semantic network analysis, including computational linguistics and text processing of SDGs in official documents, to measure interactions in specific countries (Nigeria and Spain), to be used to planners in every country. The results revealed that each SDG is connected with all the other remaining 16 SDGs, despite the language used to search for information. SDGs 2, 8, 11 and 12, known as the driving forces, were found to be always connected to all the others, and SDG5 was not among them, being translated into a difficulty in terms of transitioning from current to sustainable systems of governance and management, and failing to address the gender agenda (Rai et al., 2019).

SDG5 is clearly dependent on how governments interpret targets in order to allow women to access resources and have effective participation in all levels of societal decision-making, by involving various stakeholders in order to implement and reinforce legal and institutional arrangements on gender equality (Obura, 2020). The identification of interlinkage between the SDGs (Bali Swain & Ranganathan, 2021; Del Río Castro et al., 2021) is critical in allowing policy-makers to prioritise SDG5 targets and strategies for SD and achieving the 2030 Agenda indivisibility (Bennich et al., 2020). Biggeri et al. (2019) highlight the importance of adjusting the targets and indicators with specific goals, aiming to increase gender awareness and consciousness in the selection of parameters and to allow different strategic options to be involved in the implementation of the 2030 Agenda (Nilsson et al., 2018; Obura, 2020; Parkes et al., 2020). When assessing the sustainability performance of the Organisation for Economic Co-operation and Development (OECD) countries, Lamichhane et al. (2020) found that only 35% of OECD countries had identified a key national system to monitor all SDGs, a significant gap.

Most studies suggest that progress in achieving gender equality continues to be slow. The Global Gender Gap 2020 (World Economic Forum, 2020) report highlights the urgency of achieving gender equality, while reporting gaps between men and women in health, education and policy areas, and across all forms of economic participation, reinforcing that there is a long way to go with a 31.4% distance to parity. Women are closer than men in indicators related to health (SDG3), but further away from them in terms of employment targets. There are undoubtedly a number of local projects addressing gender equality, but it is predicted that it will take almost 100 years to close the gap in relation to political empowerment. Even in Western Europe, the same report suggests that gender equality will not be achieved for another 54 years.

Many countries are not on track to achieving the SDGs, and the COVID-19 pandemic has and continues to exacerbating widespread gender inequity (Shulla et al., 2021). Lockdowns have further increased the burden placed on women in the home and putting them at increased risk from domestic violence (Huiskes et al., 2022), with women also accounting for 70% of healthcare workers fighting the virus (UN Department of Economic & Social Affairs, 2020). In this context, and considering that the SDGs are not effectively considering gender in their implementation, the gender gap may widen, rather than narrow.

## Methodology

The work performed in the scope of this study was undertaken in three different phases:

**Phase 1**: Documenting the targets of all the 17 SDGs that would require gender issues to be accommodated before the respective SDGs can be implemented

For achieving phase 1, which also attempts to cover an information gap regarding the integration and interaction of the 17 SDGs, an effort was made to identify the main strands dominating the literature concerned with policies, aims, interactions and analytical approaches regarding SDG5 integration in the SDGs. The first step consisted in analysing how SDGs interact in the complex framework generated by the current world’s economic and social context, and therefore the methodology was based on reviewing how literature integrates gender equality leading to the UN 2030 Agenda. This resulted in a set of questions for which answers still need to be provided by considering that all SDGs need to be and are in fact interacting, guided by indivisibility, thus requiring inclusiveness as the *sine qua non* condition. Literature review allows to obtain a road overview of the existing scientific research, as well providing the context for new research (Hempel, 2020), forming the basis of all scientific research (Block & Fisch, 2020), while allowing the researcher to establish the key constructs of a future research agenda based on the identified gaps (Paul & Criado, 2020).

**Phase 2**: Presenting 16 international case studies in 13 countries that specifically reflect how gender issues are being considered when implementing the 17 SDGs

The case studies in phase 2 were selected using an open international call for collaboration, in the context of which different experts were invited to provide inputs. After a detailed and critical examination of the published research, this study allows to document the cross-cutting gender issues that should be included in the targets of each of the 17 SDGs to achieve SD, while considering SDG5. A case study was associated to each SDG, demonstrating how gender issues have been successfully infused into the actions driving the achievement of all the SDGs. Thus, by setting up the main interactions/relations and policies dominating the policy-making that addresses SDG5, and identifying current vulnerabilities, gaps and delays in this respect, the 16 international case studies reflect how gender issues are taken into account when implementing the SDGs, a necessary step in developing a judicious framework and recommendations for facilitating the achievement of SD across all SDGs, by integrating the SDG5 targets and indicators.

**Phase 3**: Develop a framework that is able to consider how gender issues across all the SDGs can be implemented to facilitate the achievement of SD at global level

In phase 3, data was first collected by documenting targets related to gender for each SDG (data from phase 1). Then, a set of case studies reflecting how gender issues have been successfully infused into the achievement of each SDG was used (data from phase 2). The combined results of both phases 1 and 2 formed the basis for the framework developed in phase 3, analysing the impact of gender issues on all the SDGs. The impact indicator showed the percentage of particular goal targets impacted by gender inequality. It was calculated for each SDG by using the following equation:

1 $$\frac{IT}{TQ}\bullet 100\%=PI$$

where *IT* represents the Impacted targets quantity, *TQ*, the total targets quantity of each goal and *PI*, the percentage impact.

The percentage values fall under one of the four categories:

- Low impact: 0%—39.9%
- Average impact: 40%—60%
- Highly impacted: 60.1%—99.9%
- Extremely impacted:100%

The combined results from the three phases are presented and discussed in the next section.

## Results and discussion

This section reports on the literature search information and data collected. The evidence collected using the case studies allowed the development of a proposed framework that can be helpful to practitioners in promoting a cross-cutting approach to gender issues in the context of all other SDGs.

### Gender equality and the SDGs

In the attempt to identify the gender issues predominant trends, the findings based on reviewing specialised literature have shown that contributions to gender equality and SDGs are mostly theoretical, focusing on trade-offs and synergies, followed by studies concerned with policy implications, and possible methodological and empirical approaches about the interactions of all the SDGs, while suggesting a wide number of indicators that are currently used or that need further refinement for properly measuring progress in achieving the SDGs. These frameworks of analyses assume particular relevance in developing countries, but also developed ones alike, as inequalities are still deeply rooted, irrespective of the SD degree.

Studies have referred to interactions among the 17 goals, while neglecting the specifics of interactions with SDG5 on gender equity studies (Abualtaher et al., 2021; Miola et al., 2019), the focus of this study. Moreover, most studies propose models and approaches often contradictory, thus delivering inconsistent outcomes regarding costs and effectiveness of policies or measures and actions for achieving the SDGs. Most of the studies are in an increasing trend of building up on the findings of other studies, while failing the novelty dimension (Magendane & Kapazoglou, 2021).

Faced with the vast volume of recent research and studies in approaching the dimension of the interaction between the SDGs, and by assessing the outcomes of relevant studies at this regard, it may be stated that most studies seek to bring improvements for three main processes: policy development, impact assessment, and how synergies are achieved or not (Alcamoet al., 2020; Biggeri et al., 2019; Scharlemann et al., 2020), while this study aims to cover both the theoretical and practical issues related to gender equity, as included in the 17 SDGs. Based on the analysed literature review, it is important to be careful about forming a generalised perspective by including general insights and gained knowledge about one SDG in relation to all other SDGs, because the context from the economic, social and environmental perspective is of paramount relevance (Nilsson et al., 2018). Integrated perspectives provide the best opportunities in assessing the relations and interactions with all other SDGs, while allowing for the identification of the main weaknesses, in particular regarding SDG5.

By affirming the overarching relevance of gender equality and its developments in the short time framework between 2015 and 2021 (Dugarova, 2018; Klasen, 2018; Odera & Mulusa, 2020), it was then possible to develop a general theoretic-empirical framework for underpinning the relevance of a gender-responsive approach to implementing the 2030 Agenda (Hirsu et al., 2018; Liu et al., 2019; Bourgault et al., 2021).

The above information does reveal the need to focus on specific practical implementation at local level, though benchmarking. The case studies presented below aim to illustrate successful implementations.

### Case Studies

Gender issues extend beyond SDG5 and needs to be addressed within all the other SDGs. The international case studies included in this section have thus been chosen as illustrative examples of gender equality, considered in relation to each SDG, other than SDG5. Further detail on how a focus on gender has brought a positive benefit in relation to each SDG, as the full list of case studies, is given in Online resource 1.

Non-governmental and governmental organisations are working together to help rural women improve the quality of their life by expanding access to sexual and reproductive health care in Tanzania (Engender Health, 2021). The Trans-Boundary Rivers of South Asia programme in Nepal promotes and supports women’s leadership in water governance to increase their social accountability (Crawford, 2020). A case study from China demonstrates that the implementation of sustainable consumption and production (SCP) may significantly benefit from the integration of gender analysis into the design of SCP policies, strengthening women’s participation in natural resource management and decision-making processes (Fan & Jaffre, 2020). In the frame of the educational programme Soochnapreneur (Information-Preneur) in India, rural women received necessary information and technology training to become change agents and assist in disseminating information regarding government schemes and benefits in communities. Participation in the programme not only develops their entrepreneurial abilities as Digital Information entrepreneurs but also allows them to charge a nominal amount for their services to sustain their livelihood (Soochnapreneur, 2021). In South Africa, the skills-driven project that supports the creation of rural, women-only entrepreneur craft groups contributes towards improving quality of life and developing a more sustainable community (Pretorius & Nicolau, 2020). The Samoa’s Ministry of Women, Community and Social Development and the Disaster Management Office are working towards increasing women’s engagement and participation in climate change and Disaster risk reduction community discussions and development projects (Aipira et al., 2017). The ‘Blue Economy Aquaculture Challenge’ initiative supports projects for transforming sustainable aquaculture practises with solutions linked to gender equality, among others (Australian Government, 2018).

The addressed case studies illustrate useful approaches for tackling a variety of local problems in a cross-cutting way, as a support for governments as they focus on gender equality issues, showing that there is room for further similar initiatives in different geographical and socio-economic contexts. The case studies presented clearly indicate that various initiatives related to gender across the globe have been successfully addressed at local levels, and these initiatives have directly and indirectly affected the achievement of the particular SDG under analysis, thus affirming the need to infuse gender issues within all the targets of the 17 SDGs to ensure more productive outcomes and achievements in the drive to SD. It has been shown that governmental and non-governmental organisations cooperate in improving the overall quality of life for women, either in rural or urban areas and in regards to health, education and access to leadership/management positions. Still, it was found that much is still to be done, as shown found below, analysing the interaction with all of the SDGs.

### The proposed framework for assessing gender equality impact across the SDGs

Achieving gender equality is a matter of human rights and is crucial to progress across all the goals and targets (Dhar, 2018), as highlighted before. Gender inequalities intersect other inequalities, power imbalance and discriminatory practices, and as such, they unequivocal serve as routes to addressing the causes preventing SD globally (Hepp et al., 2019). We have pointed out that while being a goal in its own right, gender equality cuts across all other SDGs and is reflected in 86 targets for the SDGs.

**Fig. 3:**
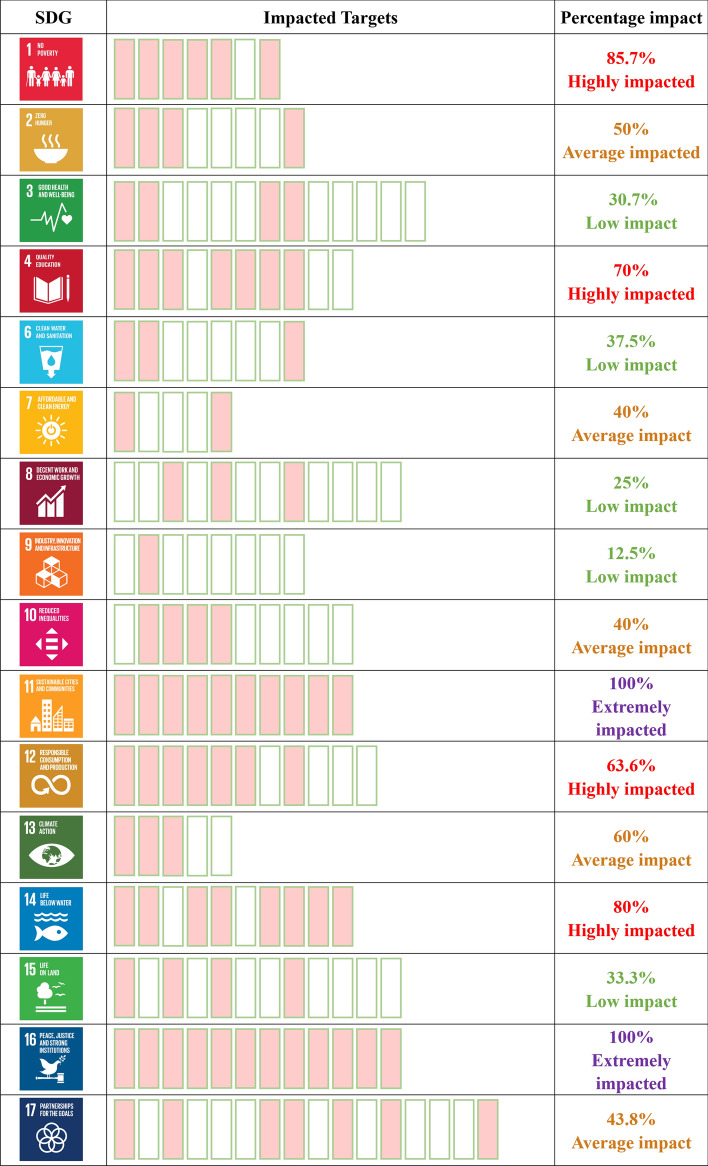
Proposed framework for considering gender impact across all the SDGs

Through the use of the data collected by documenting targets related to gender for each SDG (see the SDG Matrix–Online resource 2) and the identification of fruitful case studies reflecting how gender issues have been successfully infused into the achievement of each SDG, both based on a detailed analysis and synthesis of the literature, the authors have used the lessons learnt to develop a framework aimed at analysing the impact of gender issues on all the SDGs, illustrated in Fig. 3. This framework allows to establish which SDGs need the most attention for successful SD implementation and can serve as a guide for all practitioners in accommodating and promoting a cross-cutting approach of contemplating gender issues within the target of all the SDGs.

**Fig. 4:**
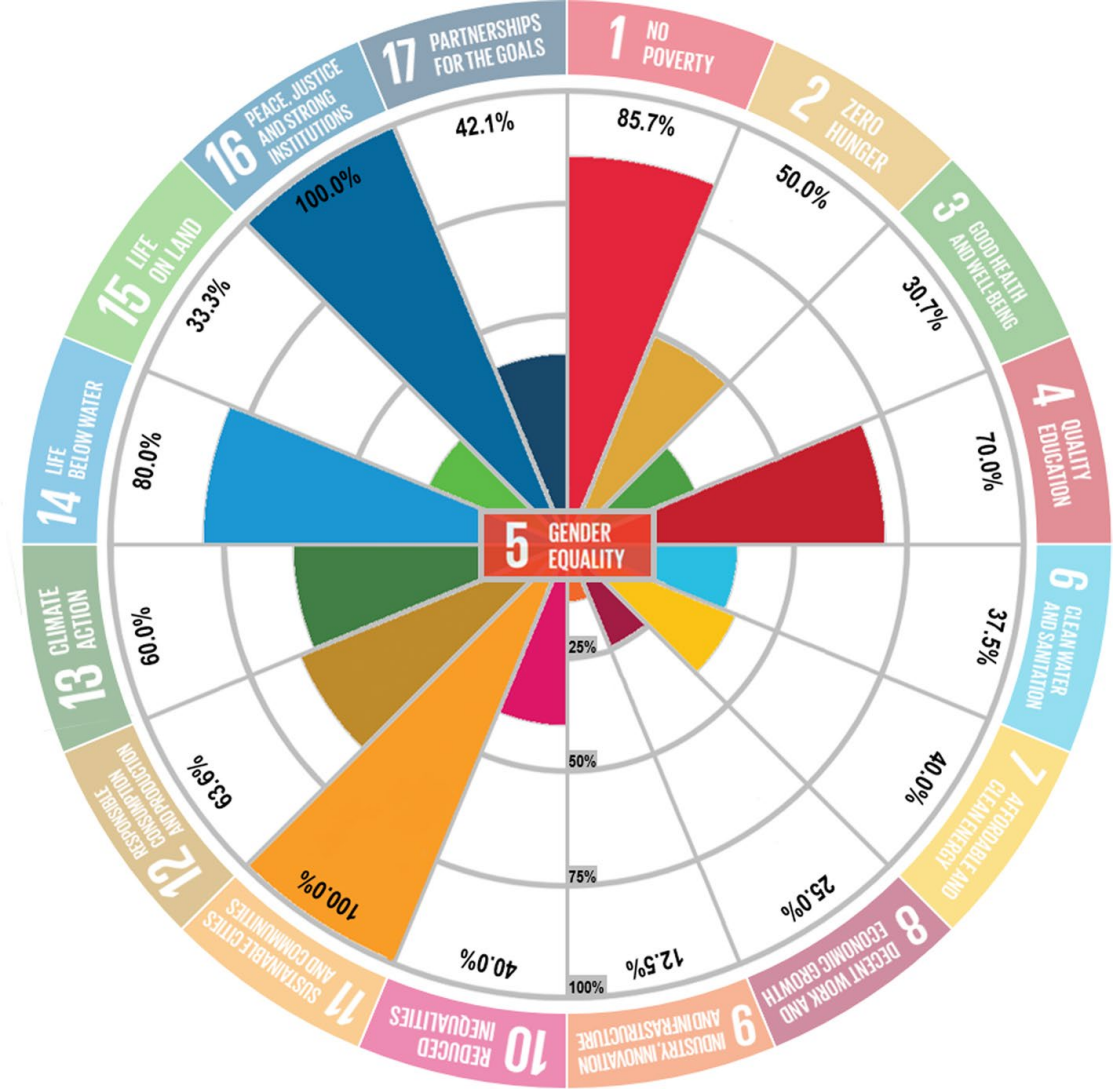
Percentage of gender inequality impact on SDG goals, according to the authors’ proposed methodology

According to the results of calculations, the following SDGs are extremely or highly impacted by gender inequality and should be prioritised: SDG1 (No Poverty), SDG4 (Quality Education), SDG11 (Sustainable Cities and Communities), SDG12 (Responsible Consumption and Production), SDG14 (Life below Water) and SDG16 (Peace, Justice and Strong Institutions) (Fig. 4). If government and non-governmental organisations strive to achieve SD, as proposed by the 2030 Agenda, they would have to ensure that gender equality is prioritised in their endeavours, particularly in the context of the six aforementioned SDGs (1, 4, 11, 12, 14 and 16).

A fundamental part of achieving SD is the reduction of poverty, and this needs greater priority in policy decisions. The literature makes it clear that high poverty is interlinked with high gender disparities (Warchold et al., 2021), particularly in developing countries (Workneh, 2020). More women are affected by poverty due to their larger share of unpaid work*,* limited access to resources and social protection, and lack of control over spending decisions when compared to men (UN, 2015). Countries that reflect statistics of more women in remunerated positions have lower poverty rates (Nieuwenhuis et al., 2018), though this might not be the case when the income size is below the poverty line (European Institute for Gender Equality, 2016). The COVID-19 pandemic is expected to have deepen gender poverty gaps, affecting women more strongly than men (Leal Filho et al., 2022b, 2022c). According to the report released by the United Nations Development Programme (UNDP) and UN Women, 232 million women will be living in extreme poverty in 2030, compared to 221 million men (Azcona et al., 2020).

Gender gaps in education negatively affect economic growth (Klasen & Lamanna, 2009). Globally, approximately 17% of women, compared to 10% of men, are illiterate. In developing countries, this gap is much larger. As example, only 26% of women are literate, compared to 46% of men in Mali, 27%, compared to 60% in South Sudan, and 70%, compared to 45% in Afghanistan (World Bank, 2020a, b). Every additional year of primary school increases the future earnings of girls, decreasing their vulnerability to violence and motivating them to marry later (UN Women, 2012). Addressing gender imbalance in land ownership rights and access to natural, social and economic resources is essential for responsible consumption and production (Franco et al., 2018). Women demonstrate a higher tendency towards product reuse, waste reduction, and purchase of organic and eco-labelled products (Bulut et al., 2017; OECD, 2018). The promotion of peaceful and inclusive societies for SD and access to justice for all are impossible without targeting gender inequalities. In 2020, the United Nations High Commissioner for Refugees (UNHCR) recorded more than 82 million people fleeing war, violation of human rights, persecution or conflict, of which 48% are women and girls (UNHCR, 2021). The COVID-19 pandemic and subsequent lockdowns have intensified domestic violence (Azcona et al., 2020; UN Women, 2020b; Akel et al., 2021; Bourgault et al., 2021). The preliminary data indicate a 25%—100% increase in reported cases globally (UN Women, 2020c), one of the consequences of the inability of institutions to provide equal gender access to justice and essential services, and of gender representation imbalance in global, regional or national governance (UN Women, 2018). Particularly in developing countries, the achievement of the social inclusion of vulnerable groups such as women can be ensured by local government policies, especially related to well-being gender budgeting (Gunluk-Senesen, 2021). More equal gender participation is one of the key factors to sustainable peace.

## Conclusions

A recent major challenge impairing the proper achievement of gender equality is the COVID-19 pandemic, which is causing an expansion of inequalities in topics related to education, employment and well-being, healthcare, consumption and production, or climate change, being imperative that all stakeholders involved in SD thus prioritise and infuse gender equality in all their endeavours, while policy-makers need to critically reflect on whether their strategies for particular individual goals would be enhanced by a broader consideration of gender equality issues. While most of the previous studies investigated the potential interactions of gender equality with other SDGs (Barbier & Burgess, 2019; Dawes, 2022; Pham-Truffert et al., 2020; Tremblay et al., 2020; van Zanten & van Tulder, 2021; Warchold et al., 2021), this study contributes to a better understanding of gender equality as a cross-cutting issue among all the SDGs, underscoring the need to prioritise gender issues at all scopes of SD.

This study aimed to assess and define the relations and interactions regarding gender inequality, based on specific literature related to main gender inequality concerns, access to education, employment and implicitly to equal pay, along with all other related issues, from legal aspects to metrics of violence. An extensive body of literature was explored in this study, also documenting 16 relevant international case studies in 13 countries to emphasise the significance of positive interventions in terms of gender equality, considered as a cross-cutting issue among all the other SDGs, as reflected in 86 targets. As a result, the study proposes an innovative qualitative assessment framework, according to which targets can be impacted negatively by gender inequality, an important factor that can impair the achievement of a particular SDG. Among the most-impacted SDGs that should more attentively consider the promotion of gender equality as an important condition for their achievement are SDGs 1, 4, 11, 12, 14 and 16, being possible to notice a strong diversity of approaches involved, covering issues of concern that are equally of future interest. Understanding the strong interconnectedness of the SDGs in terms of addressing the issues related to gender equality needs to become a trend. If widely spread, this trend may serve as an accelerator for the achievement of global SD, through the 17 SDGs, and can offer further guidance to policy-makers for prioritising the achievements of the targets, by empowering women worldwide. The literature review outlines that the progress in achieving gender equality continues to be slow, as many gaps still exist between men and women in health, education, politics, and across all forms of economic participation. However, as demonstrated by the successful case studies implemented worldwide, there is a growing interest among different stakeholders to develop collaborative initiatives that give particular attention to promoting gender equality, and the trend is likely to increase in the future. However, while the presented case studies illustrate positive interventions in terms of SDG5 contribution to SD, they are clearly still insufficient.

One all-encompassing finding is that in spite of a wide range of studies and academic papers related to SDGs and SD, there continues to be divisiveness in assessing the challenges and opportunities of the 2030 Agenda, associated with the need for developing sound frameworks for drafting and assessing *ex-ante* policies, measures and actions for ensuring the integrated interaction among the 17 SDGs, by considering necessary trade-offs and integrating other environmental, social and economic policy objectives. All these, while not explicitly mentioned in this study, have been implicitly considered, along with policy paradigms that consider the lifestyle, technological and even healthcare/educational changes. The 17 SDGs of the 2030 Agenda imply by their formulation a principle of indivisibility, as SDGs address the shared concerns of all humanity. In fact, it is precisely this governing principle which is the foundation for the approach used in this study, guided by the interest in analysing how SDG5 can be assessed and further implemented when associated to the other 16 SDGs, substantiated by the fact that the 2030 Agenda has an implied target-integrated approach regarding the SDGs. Investigating SDG5 relationship with the other 16 SDGs proved to be challenging and promising, as it provided for new insights about the relationships and interactions between all the SDGs. Thus, a key implication of this study is that it illustrates the fact that more attention should be given to mainstreaming the gender equality theme within all development initiatives of every country. Also, considerations to gender issues should be included in the design of targeted policies and programmes, data collection on indicators, and also in the defining of priorities in every region.The study has limitations. The first one is the fact that, being a qualitative study, it was not possible to cover all the works published in the field. Also, the selection of the case studies was not exhaustive or intended to cover all geographical regions, and it should be only regarded as an illustration of gender equality as a cross-cutting issue. Furthermore, the sample of 13 countries does not cater for a worldwide representation. However, despite these limitations, this study represents a significant knowledge addition to the existing literature on the connections between SDG5 and overall efforts to implement global SD and successfully advancing the SDGs.

Based on the evidence collected, the following **recommendations** may help in efforts aimed at placing matters related to gender more centrally in the delivery of the SDGs:

- Inclusion of gender issues as a cross-cutting topic in the implementation of the SDGs.
- A greater emphasis on gender equality in SDGs-related projects across all themes.
- An increased attention should be paid to the opinion, views and voices of women on SDGs-associated policies, a procedure often overlooked.
- More attention should be given to poverty alleviation, a trend often unnoticed in gender discussions.
- A more detailed and continued review of novel case studies across the globe should be undertaken to establish how existing good practices on mainstreaming gender are integrated into the targets of all the SDGs, and then to infuse these local initiatives into policy and development initiatives.

Finally, there is a perceived need to build more capacity among professionals involved in the implementation of the SDGs, so as to better sensitise them about the need to always consider gender issues, raising global awareness about gender-related matters.

## Supplementary Information

Below is the link to the electronic supplementary material.

Supplementary file1 (PDF 194 kb)

Supplementary file2 (PDF 725 kb)

## Data Availability

The manuscript has data included as electronic supplementary material.
