# Supplementary material for: Promoting gender equality across the sustainable development goals
Source: Environ Dev Sustain. 2022 Sep 15:1–22. Online ahead of print. doi: 10.1007/s10668-022-02656-1 (PMC9476407; doi:10.1007/s10668-022-02656-1)
Supplement: Supplementary file 1 — Supplementary file1 (PDF 194 kb) [file 10668_2022_2656_MOESM1_ESM.pdf]

Online Resource 1- International case studies illustrating worldwide initiatives that address gender equality among the SDGs

Country	SDG	Case study description	References
International	SDG 1 (No poverty)	Study focusing on "gendered poverty." The study's main aim is on the UN Women and the progress of women in the years 2015-2016, acknowledging that gendered poverty is a consequence of gender inequality.	Bradshaw et al., 2017
India	SDG 2 (Zero hunger)	Study documenting the relationship between gender inequality and hunger in India. 95 million (2.5% global hunger burden) undernourished people exist. The most vulnerable are women and children. Malnutrition has more effect on women's and girls' health, at a greater risk of giving birth to low-birth weight infants.	Callister, 2018

Tanzania	SDG 3 (Good health and well-being)	Engender Health Tanzania is helping women accessing to reproductive healthcare. With a population of nearly 56.32 million, many Tanzanian women,do not have access to contraceptives, HIV testing/counselling services and health facilities to deliver babies. Since 1982, EngenderHealth has been working with the government, contributing to improving healthcare.	Engender Health, 2021
India	SDG 4 (Quality Education)	Soochnapreneur (Information-Preneur) is a unique project focusing on education about the different government schemes and citizen's rights for people in rural and remote parts of India. Bridging the digital-divide, young rural women and youth, acting as Information-Preneurs, provide the required information to those who have no access to technology, also helping them to generate some small amount of money through information services. 100 Information-Preneurs have assisted more than 7,000 rural people. Many women participate in the project, benefitting directly by earning as Information-Preneurs and indirectly through access to the different government schemes.	Soochnapreneur, 2021<br>Qualcomm Wireless Reach, 2018

Nepal	SDG 6 (Clean water and sanitation)	Study related to the Trans-Boundary Rivers of South Asia (TROSA) programme, an opportunity for women’s leadership in water governance to increase social accountability. Women are sensitised on their rights regarding riverine water resource planning and decision-making, advocating for key water governance issues through service providers and local government.	Crawford, 2020
India	SDG 7 (Affordable and clean energy)	Using India as an illustrative case, a mixed-methods study was conducted. Women are neither the sole nor primary beneficiaries of electricity access, even when appliances that would particularly benefit them are affordable. While energy access could improve gender equity, intra-household power dynamics were highlighted as an important boundary condition in realising more equitable energy access.	Rosenberg et al., 2020
Turkey	SDG 8 (Decent work and economic growth),	Study indicating that a fiscal prioritisation on building a social infrastructure of care, greater than investments in physical infrastructure/construction or cash transfers, presents an enormous potential for decent job creation, particularly in the female-dominated occupations and sectors, promoting gender equality and better access to decent work.	Ilkkaracan et al., 2015

Comparison France, Spain, Morocco and Algeria	SDG 9 (Industry innovation infrastructure)	Research focusing on the importance of women’s participation as a key element in achieving the SDGs, by analysing the existing digital gender gap with the achievement of the SDGs, concluding that equal access to digital technologies is important to achieving all the SDGs.	Kerrasm et al., 2020
Philippines	SDG 10 (Reducing inequality)	Research documenting gender-related indicators that can be used to monitor gender equality and women empowerment. An overview of equality of human capabilities, equality of economic opportunity, equality in political voice and leadership, and the safety of women and girls is provided. Priorities for public policy involving gender transformational issues to attain equality and women's empowerment are given.	David et al., 2018
South Africa	SDG 11 (Sustainable cities and communities)	Skills-driven project enabling a rural community to drive changes that will improve quality of life. The creation of a female-only entrepreneur craft group is included. The craft group initiative gives the group of women the opportunity to contribute meaningfully towards a collective drive to a more sustainable community.	Pretorius and Nicolau, 2020
Peoples Republic of China	SDG 12 (Responsible consumption and production)	Study focusing on gender awareness in sustainable consumption and production (SCP). Research concludes that integrating gender analysis into the design of SCP policies, addressing remaining gender gaps, and strengthening women’s participation in natural resource management and decision-making can have a positive effect and support the shift towards SCP.	Fan and Jaffre, 2020

Pacific Countries	SDG 13 (Climate action)	Study focusing on the findings of research by United Nations (UN) Women 2015 to investigate the connection between gender and climate change in the Pacific Region. The study provides evidence-based information about the gender impact of climate change, how the gender inequalities are directed by climate change adaptation and how to enhance women empowerment.	Aipira et al. 2017
Australia	SDG 14 (Life below water)	For implementing SDGs 14, Australia funded the “Blue Economy Aquaculture Challenge” initiative, in order to support, projects for transforming sustainable aquaculture practices. Many of the solutions promised outcomes linked to other SDGs, such as gender equality (SDG5), health and nutrition (SDG3) employment, and SDP (SDG 12).	Australian Government, 2018
Latin American countries	SDG 15 (Life on land)	Research focusing on women’s unequal access to land and the proposed indicators to measure progress. The study in Latin America demonstrates the current degree of inequality in the gender distribution of landholders and landowners, and why it is important that countries improve gender statistics, collecting gender disaggregated data on both land ownership and agricultural decision-making.	Deere, 2018
Australia	SDG 16 (peace, justice and strong institutions)	The Australian government supports global peace and justice, by acting as a global leader for advancing commitments to the Women, Peace and Security Agenda. Considerable work was done to integrate a gender perspective into international peace and security policy outcomes, training Australian	Australian Government, 2018

		personnel to safeguard the needs of women in conflict zones.	
Australia	SDG 17 (Partnerships for the goal)	The Australian government, in partnership with the Australian National University and the International Women's Development Agency, has introduced the Individual Deprivation Measure (IDM), a gender-sensitive, multidimensional and below household level (individual level). It aligns with 25% of the 53 gender-related SDG Indicators and identifies gender sensitive deprivation, in order to address poverty.	Australian Government, 2018

Source: Authors' elaboration

References

Aipira, C., Kidd, A., & Morioka, K. (2017). Climate Change Adaptation in Pacific Countries: Fostering Resilience Through Gender Equality. In W. Leal Filho, (Ed.), *Climate Change Adaptation in Pacific Countries. Climate Change Management.* Springer, Cham. https://doi.org/10.1007/978-3-319-50094-2_13

Australian Government (2018). *Report on the implementation of the sustainable Development goals*. https://www.sdgdata.gov.au/sites/default/files/voluntary_national_review.pdf

Bradshaw, S., Chant, S., & Linneker, B. (2017). Gender and poverty: what we know, don't know, and need to know for Agenda 2030. *Gender, Place & Culture*, *24*(12), 1667-1688. https://doi.org/10.1080/0966369X.2017.1395821

Callister, L. C. (2018). Reducing Hunger Among Women and Children in India. *MCN American Journal of Maternal/Child Nursing,* *43*(4), 234. https://doi.org/10.1097/NMC.0000000000000445

Crawford, E. (2020). Achieving Sustainable Development Goas 5 and 6: The case for gender-transformative water programmes. Oxfam, Oxford, UK. https://doi.org/10.21201/2020.5884

David, C. C., Ramon, J. Albert, G., & Vizmanos, J. (2018). Sustainable Development Goal 5: How Does the Philippines Fare on Gender Equality? Research Paper Series No. 2018-04, Philippine Institute for Development Studies. Quezon City.

Deere, C. D. (2018). Sustainable Development Goals, Gender Equality and the Distribution of Land in Latin America. Cadernus Pagu, 52. https://doi.org/10.1590/18094449201800520006.

Engender Health (2021). *Tanzania*. https://www.engenderhealth.org/our-countries/africa/tanzania/

Fan, L., & Jaffre, V. N. (2020). *The gender dimension of sustainable consumption and production: A micro survey-based analysis of gender differences in awareness, attitudes, behaviours in the People Republic of China*. Asian Development Bank, Hong Kong.

Ilkkaracan, I., Kim, K., & Kayaaugust, T. (2015). The Impact of Public Investment in Social Care Services on Employment, Gender Equality, and Poverty: The Turkish Case. İstanbul Technical University Women's Studies Center in Science, Engineering and Technology and the Levy Economics Institute of Bard College.

Kerrasm, H., Sanchez-Navarro, J., Lopez-Becerra, E. I., & de-Miguel Gomez, M. D. (2020). *Sustainability*, *12*, 3347.

Pretorius, R. W., & Nicolau, M. D. (2020). Empowering communities to drive sustainable development: Reflections on experiences from rural South Africa. In: W. Leal Filho, U. Tortato, & F. Frankenberger (Eds.), *Universities and Sustainable Communities:*

Meeting the goals of the Agenda 2030 (pp. 529-545). Springer, Cham. https://doi.org/10.1007/978-3-030-30306-8-32

Qualcomm Wireless Reach (2018). Soochnapreneur. https://www.qualcomm.com/media/documents/files/india-soochnapreneur-program.pdf

Rosenberg, M., Armanios, D. E., Aklin, M., & Jaramillo, P. (2020). Evidence of gender inequality in energy use from a mixed study in India. *Nature Sustainability*, *3*, 110-118.

Soochnapreneur (2021). *About the Programme*. https://soochnapreneur.in/about-the-programme/
