# Supplementary material for: Promoting gender equality across the sustainable development goals
Source: Environ Dev Sustain. 2022 Sep 15:1–22. Online ahead of print. doi: 10.1007/s10668-022-02656-1 (PMC9476407; doi:10.1007/s10668-022-02656-1)
Supplement: Supplementary file 2 — Supplementary file2 (PDF 725 kb) [file 10668_2022_2656_MOESM2_ESM.pdf]

Online resource 2– SDG Matrix

SDG Number and Target	Target description	Implications	References
**SDG 1 End poverty in all its forms everywhere**			
**Target 1.1**	By 2030, eradicate extreme poverty for all people everywhere, currently measured as people living on less than $1.25 a day	Women and the most vulnerable in society are for the most part more impacted by poverty. As such, ensuring the eradication of extreme poverty will benefit more women and help end and/or reduce poverty. Extreme poverty and its impact on women is exacerbated by discriminatory social norms that alienate women when it comes to equal opportunities in education as well as employment and as such leads to those affected living on less than $1.25 a day in mostly the developing world.	Olinto et al., 2013<br>Lang & Lingnau, 2015<br>Franco & Minnery, 2020
**Target 1.2**	By 2030, reduce at least by half the proportion of men, women and children of all ages living in poverty in all its dimensions according to national definitions	Strong and robust policies can play a role in reducing poverty. For example, as poverty takes many forms including that of energy, a consideration of place of residence, house ownership status, family size, and the age of the primary breadwinner can mitigate against multidimensional energy poverty and play a significant role in national definitions of poverty. Such considerations could have an impact on poverty reduction in at least half of the proportion of men, women and children in the most deprived areas of society.	Abbas et al., 2020

**Target 1.3**	Implement nationally appropriate social protection systems and measures for all, including floors, and by 2030 achieve substantial coverage of the poor and the vulnerable	Social security is an important human right which is central to the reduction of poverty, achieving equality and avoiding social exclusion. These aspects allow for the promotion of equal opportunities including gender equality, which if properly implemented can assist with the development of appropriate social security systems and measures that could have a positive impact for both men and women.	International Labour Organisation, 2012<br>Kaltenborn, 2017
**Target 1.4**	By 2030, ensure that all men and women, in particular the poor and the vulnerable, have equal rights to economic resources, as well as access to basic services, ownership and control over land and other forms of property, inheritance, natural resources, appropriate new technology and financial services, including microfinance	Land and resource tenure, including inheritance, access to and use of technology, financial services, and access to microfinance are important for meeting SDG targets such as the one in question. For example, when it comes to land ownership, because of unequal rights to land, there ought to be explicit protection of land rights that are recognised by both customary and statutory law for women, which can allow them to fairly participate in land ownership, management and administration.<br>Furthermore, microfinance gives women the opportunity to empower themselves and improve their lives. This has the ability to remove them from poverty.	Katila et al., 2020<br>Hansen et al., 2020
**Target 1.5**	By 2030, build the resilience of the poor and those in vulnerable situations and reduce their exposure and	Technical, policy, capacity enhancement and finance that are among the elements of transformative approaches in agriculture and food security will be necessary to tackle climate change. In particular, because women are most impacted by poverty and	Campbell et al., 2018

	vulnerability to climate-related extreme events and other<br><br>economic, social and environmental shocks and disasters	hunger, climate change has a direct impact on them in this respect.	
**Target 1.b**	Create sound policy frameworks at the national,<br><br>regional and international levels, based on pro-poor and<br><br>gender-sensitive development strategies, to support<br><br>accelerated investment in poverty eradication actions	The participation of women in policy making and the holding of high office such as parliamentary seats has a positive impact on women's well-being, including girls' education, fertility, child and infant mortality, and early marriage	Konte, 2020
**SDG 2 End hunger, achieve food security and improved nutrition and promote sustainable agriculture**			
**Target 2.1**	By 2030, end hunger and ensure access by all people,<br><br>in particular the poor and people in vulnerable situations,<br><br>including infants, to safe, nutritious and sufficient food all year-round.	It is well understood that due to gender inequalities, women and the more vulnerable in society will be more affected by hunger. Due to evident societal gender inequalities resulting from social, political and economic marginalisation of women, such inequalities have an impact on food security and resulting hunger, not only for women but for infants as well. With hunger come issues around health and the inability, as well as a lack of capability, to be able to effectively contribute to sustainable development in society	Larson & Larson, 2019

**Target 2.2**	By 2030, end all forms of malnutrition, including<br><br>achieving, by 2025, the internationally agreed targets on stunting and wasting in children under 5 years of age, and address the nutritional needs of adolescent girls, pregnant and lactating women and older persons	Women are more likely to be overweight and obese. In addition, one in three women of reproductive age suffer from anaemia. This has implications for children as well as pregnant and lactating women, due to the lack of nutrients that such ailments have. As such, it is imperative that nutrition takes into consideration healthy and sustainable dietary needs when looking to end all forms of malnutrition. Healthy, nutritional and sustainable diets will need to be considered into ethically thought-out national policies that cover issues around taxes, incentives, nudges, and subsidies, especially for those most affected.<br><br>Furthermore, encouraging indigenous food systems in traditional communities can play a role in sustainable nutritional diets that can help end malnutrition. When it comes to the developing of national policies around and/or encouraging good dietary consumption of indigenous food systems, mothers, who are usually responsible for family food and meals, should be a targeted priority.	Fanzo, 2019<br><br>Ghosh-Jerath et al., 2020
**Target 2.3**	By 2030, double the agricultural productivity and<br><br>incomes of small-scale food producers, in particular women, indigenous peoples, family farmers, pastoralists and fishers, including through secure and equal access to	Equal participation of all relevant stakeholders, which should include women in agricultural productivity, will result in increased productivity. In particular, development policies should not only favour urban industrial areas but should be inclusive of agricultural and rural settings where there might be more women inhabiting such spaces in order to increase agricultural	Rodríguez-Pose and Hardy, 2015

	land, other productive resources and inputs, knowledge, financial services, markets and opportunities for value addition and non-farm employment	productivity for sustainable development that could help end hunger.	
**Target 2.c**	Adopt measures to ensure the proper functioning of food commodity markets and their derivatives and facilitate timely access to market information, including on food reserves, in order to help limit extreme food price volatility	Without a consideration for gender inequalities, particularly when it comes to recognising the need for information on food commodity markets and the fact that most small, medium-sized and family farms are run by women, it might prove a challenge to have proper functioning food commodity markets. Therefore, measures and policies that consider necessary policy measures that address and acknowledge this phenomenon will go a long way in achieving the target in question.	Fontefrancesco, 2019
**SDG 3 Ensure healthy lives and promote well-being for all at all ages**			
**Target 3.1**	By 2030, reduce the global maternal mortality ratio to less than 70 per 100,000 live births	Equalising the social and economic status of women would improve the women's health outcomes	Abebe, 2016
**Target 3.2**	By 2030, end preventable deaths of new-borns and children under 5 years of age, with all countries aiming to reduce neonatal mortality to at	Giving indistinguishable preference to all genders will ensure child survival with the reduction in excess male over female child mortality	Iqbal et al., 2018

	least as low as 12 per 1,000 live births and under-5 mortality to at least as low as 25 per 1,000 live births		
**Target 3.7**	By 2030, ensure universal access to sexual and reproductive health-care services, including for family planning, information and education, and the integration of reproductive health into national strategies and programmes.	Giving equal opportunity and easy accessibility to formal and quality education to females will impact by generating responsible attitudes towards health	Queen Mary University of London, 2018
**Target 3.8**	Achieve universal health coverage, including financial risk protection, access to quality essential health-care services and access to safe, effective, quality and affordable essential medicines and vaccines for all.	Inclusion of women in economic and decision-making activity will increase the ability to access existing maternal and child health services	Singh et al., 2015
**SDG 4 – Ensure access to inclusive and equitable quality education for boys and girls, from pre-primary through to secondary education, and promote lifelong learning opportunities for all**			
**Target 4.1**	By 2030, ensure that all girls and boys complete free, equitable and quality primary and secondary education	Education of girls is also important for the achievement of a sustainable future.	Herbert et al., 2020<br>Spiteri, 2020

	leading to relevant and effective learning outcomes		Spiteri, 2018<br><br>UNESCO, 2018a<br><br>Stromquist, 2020
**Target 4.2**	By 2030, ensure that all girls and boys have access to quality early childhood development, care and pre-primary education so that they are ready for primary education	All children need good-quality early childhood education, which sets the foundations for lifelong learning. It helps to improve their future outcomes and alleviate poverty.	
**Target 4.3**	By 2030, ensure equal access for all women and men to affordable and quality technical, vocational and tertiary education, including university	Lifelong education for women is important at every stage of life.	
**Target 4.5**	By 2030, eliminate gender disparities in education and ensure equal access to all levels of education and vocational training for the vulnerable, including persons with disabilities, indigenous peoples and children in vulnerable situations	Women should have equal access to education to benefit from future employment prospects.	

**Target 4.6**	By 2030, ensure that all youth and a substantial proportion of adults, both men and women, achieve literacy and numeracy	The education of women is important for the achievement of a sustainable society.	
**Target 4.7**	By 2030, ensure that all learners acquire the knowledge and skills needed to promote sustainable development, including, among others, through education for sustainable development and sustainable lifestyles, human rights, gender equality, promotion of a culture of peace and non-violence, global citizenship and the appreciation of cultural diversity and of culture's contribution to sustainable development	Education, and education for sustainable development (ESD) in particular, help promote gender equality and human rights, thus encouraging children and adults to become ecologically responsible citizens. Therefore, barriers to access to education need to be overcome by governments to ensure quality education for all and eliminate gender disparities.	
**Target 4.A**	Build and upgrade education facilities that are child, disability and gender sensitive and provide safe, nonviolent,	Girls and boys with disabilities have a right to good quality education that promotes sustainable behaviours in children.	

	inclusive and effective learning environments for all		
**SDG 6 Ensure availability and sustainable management of water and sanitation for all**			
**Target 6.1**	By 2030, achieve universal and equitable access to safe and affordable drinking water for all.	Proportion of population using safely managed drinking water services	Freistein and Mahlert, 2015
**Target 6.2**	By 2030, achieve access to adequate and equitable sanitation and hygiene for all and end open defecation, paying special attention to the needs of women and girls and those in vulnerable situations.	Proportion of population using (a) safely managed sanitation services and (b) a hand-washing facility with soap and water	Nygård, 2017
**Target 6.b**	Support and strengthen the participation of local communities in improving water and sanitation management	Proportion of local administrative units with established and operational policies and procedures for participation of local communities in water and sanitation management	Klarin, 2018
**SDG 7 Ensure access to affordable, reliable, sustainable and modern energy for all**			
**Target 7.1**	By 2030, ensure universal access to affordable, reliable and modern energy services.	Proportion of population with access to electricity	

**Target 7.b**	By 2030, expand infrastructure and upgrade technology for supplying modern and sustainable energy services for all in developing countries, in particular least developed countries, small island developing States and landlocked developing countries, in accordance with their respective programmes of support	International financial flows to developing countries in support of clean energy research and development and renewable energy production, including in hybrid systems.<br><br>Installed renewable energy-generating capacity in developing countries (in watts per capita)	Bhandari & Shvindina, 2019
**SDG 8 Promote sustained, inclusive and sustainable economic growth, full and productive employment and decent work for all**			
**Target 8.3**	Promote development-oriented policies that support productive activities …access to financial services.	SDG 8 promotes "sustained, inclusive and sustainable economic growth, full and productive employment and decent work for all". Businesses play a key role for job creation and economic growth. Instituting non-discriminatory practices and embracing diversity and inclusion will also lead to greater access to skilled, productive talent.	Dugarova, 2018<br><br>Pandey & Kumar, 2019<br><br>Buhmann et al., 2019<br><br>Rai et al., 2019<br><br>Manandhar et al., 2018<br><br>Alarcón and Cole, 2019
**Target 8.5**	By 2030, achieve full and productive employment and	SDG 8 promotes 'sustained, inclusive and sustainable economic growth, full and productive employment and	

	decent work for all women and men, including for young people and persons with disabilities, and equal pay for work of equal value.	decent work for all’. Businesses play a key role for job creation and economic growth. Instituting non-discriminatory practices and embracing diversity and inclusion will also lead to greater access to skilled, productive talent.	
**Target 8.8**	Protect labour rights…and those in precarious employment	The main objective is to achieve an inclusive employment.<br>Protection of labour rights by enhancing health and safety at work.<br>Key themes: economic inclusion; non-discrimination	
**SDG 9 Build resilient infrastructure, promote inclusive and sustainable industrialization and foster innovation**			
**Target 9.2.**	Promote inclusive and sustainable industrialization and, by 2030, significantly raise industry’s share of employment and gross domestic product, in line with national circumstances, and double its share in least developed countries.	Increasing investments in infrastructure has positively affected gender equality by reducing unpaid care and domestic work.<br>Better infrastructure can improve health and education for women and children.<br>Women’s access to education and employment contributes to industrialisation.<br>Investing in social infrastructure could increase employment both in emerging countries and in developed economies.	Sinha et al., 2020

		Reducing gender gap and subject segregation in education (by increasing gender diversity in STEM) can boost innovations.	
**SDG 10 Reduce inequality within and among countries**			
**Target 10.2**	By 2030, empower and promote the social, economic and political inclusion of all, irrespective of age, sex, disability, race, ethnicity, origin, religion or economic or other status	Improving the labour market conditions by promoting decent work and equal employment opportunities to all genders	Abebe, 2016
**Target 10.3**	Ensure equal opportunities and reduce inequalities of outcome, including by eliminating discriminatory laws, policies and practices and promoting appropriate legislation, policies and action in this regard	Promoting equal pay for equal work, irrespective of gender, will encourage the suppressive gender to join the workforce, enjoying equal status	Abebe, 2016
**Target 10.4**	Adopt policies, especially fiscal, wage and social protection policies, and progressively achieve greater equality	Reinforce labour laws, including minimum wages, prevention of sexual harassment of women at the workplace, decent work, freedom of association and collective bargaining	United Nations System Chief Executives Board for Coordination, 2017
**Target 10.5**	Improve the regulation and monitoring of global financial markets and institutions and	Promoting transparency and accountability of private companies to reduce inequalities among different genders	SDG Compass, 2021

	strengthen the implementation of such regulations		
**SDG 11 Make cities and human settlements inclusive, safe, resilient and sustainable**			
**Target 11.1**	By 2030, ensure access for all to adequate, safe and affordable housing and basic services and upgrade slums	Proportion of urban population living in slums, informal settlements or inadequate housing	Lucci et al., 2015<br>Nzau & Trillo, 2020<br>Nabutola, 2004<br>Hoek-Smit et al., 2020<br>JLL, 2016<br>Chattopadhyay et al., 2016<br>Kaur, 2018<br>Teotia, 2015<br>Nallathiga, 2019<br>Arimah, 2001<br>Santoro, 2015<br>Gopalan & Venkataraman, 2015<br>Woetzel et al., 2014

**Target 11.2**	By 2030, provide access to safe, affordable, accessible and sustainable transport systems for all, improving road safety, notably by expanding public transport, with special attention to the needs of those in vulnerable situations, women, children, persons with disabilities and older persons	Proportion of population that has convenient access to public transport, by sex, age and persons with disabilities	Kett et al., 2020<br><br>European Institute for Gender Equality, 2020<br><br>Leach, 2015<br><br>UN Women, 2018a<br><br>Liu et al., 2020<br><br>ESCAP, 2017
**Target 11.3**	By 2030, enhance inclusive and sustainable urbanization and capacity for participatory, integrated and sustainable human settlement planning and management in all countries	Ratio of land consumption rate to population growth rate	Blei et al., 2018<br><br>Agyemang &<br><br>Morrison, 2017<br><br>United Nations Environment Programme, 2017<br><br>European Environment Agency, 2015<br><br>Desai, 2020<br><br>Mansell et al., 2020<br><br>Abdulkadir et al., 2019<br><br>Nicolau et al., 2019

**Target 11.4**	Strengthen efforts to protect and safeguard the world's cultural and natural heritage	Total expenditure (public and private) per capita spent on the preservation, protection and conservation of all cultural and natural heritage.	Wang et al., 2018<br>Satterthwaite, 2017<br>Smiciklas et al., 2017<br>Idowu et al. (eds), 2020<br>Sterling, 2016<br>Seto et al., 2014<br>OECD, 2018
**Target 11.5**	By 2030, significantly reduce the number of deaths and the number of people affected and substantially decrease the direct economic losses relative to global gross domestic product caused by disasters, including water-related disasters, with a focus on protecting the poor and people in vulnerable situations	Number of deaths, missing persons and directly affected persons attributed to disasters per 100,000 population	International Council for Science, 2011<br>Smas et al., 2013<br>Hoornweg & Pope, 2017
**Target 11.6**	By 2030, reduce the adverse per capita environmental impact of cities, including by paying special attention to air quality and municipal and other waste management	Proportion of urban solid waste regularly collected and with adequate final discharge out of total urban solid waste generated, by cities	United Nations, 2018<br>Ros-Tonen et al., 2016<br>Keivani, 2010

			Overseas Development Institute, 2018
**Target 11.7**	By 2030, provide universal access to safe, inclusive and accessible, green and public spaces, in particular for women and children, older persons and persons with disabilities	Provide universal access to safe, inclusive and accessible green and public spaces by 2030.	Damodaran et al., 2015<br>Devisscher et al., 2019<br>Guedes Vidal et al, 2019<br>Santiago Pineda et al., 2017<br>DIAUD/ CBM, 2016<br>Daniel, 2016
**Target 11. 8**	Support positive economic, social and environmental links between urban, peri-urban and rural areas by strengthening national and regional development planning	Proportion of population living in cities that implement urban and regional development plans integrating population projections and resource needs, by size of city.	Grogan, 2020<br>Clos, 2016<br>Cohen, 2006
**Target 11.9**	By 2020, substantially increase the number of cities and human settlements adopting and implementing integrated policies and plans towards inclusion, resource efficiency,	Substantially increase the number of cities and human settlements adopting and implementing holistic disaster risk management at all levels	United Nations Statistics Division, 2016

	mitigation and adaptation to climate change, resilience to disasters, and develop and implement, in line with the Sendai Framework for Disaster Risk Reduction 2015-2030, holistic disaster risk management at all levels		
**Target 11.10**	Support least developed countries, including through financial and technical assistance, in building sustainable and resilient buildings utilizing local materials	Proportion of financial support to the least developed countries that is allocated to the construction and retrofitting of sustainable, resilient and resource-efficient buildings utilising local materials	The Economist Intelligence Unit, 2019<br>Norichika & Biermann, 2017<br>Servaes, 2017
**SDG 12 Ensure sustainable consumption and production patterns**			
**Target 12.1**	Implement the 10-year framework of programmes on sustainable consumption and production, all countries taking action, with developed countries taking the lead, taking into account the	Gender issue is underestimated when it comes to sustainable production and consumption. In general SDG 12 has been considered 'gender blind'.	Herbert et al., 2020<br>UN Women, 2020a<br>Pudaruth et al., 2015<br>Cho et al., 2018

	development and capabilities of developing countries		
**Target 12.2**	By 2030, achieve the sustainable management and efficient use of natural resources	Women are under-represented in decision-making related to sustainable management and efficient use of natural resources, so it is important to increase their participation in leadership and decision–making processes, for the sustainable management of production and consumption patterns	
**Target 12.3**	By 2030, halve per capita global food waste at the retail and consumer levels and reduce food losses along production and supply chains, including post-harvest losses	Women have an important role in achieving this goal, however the recognition and actions to address this are rather limited still.	
**Target 12.4**	By 2020, achieve the environmentally sound management of chemicals and all wastes throughout their life cycle, in accordance with agreed international frameworks, and significantly reduce their release to air, water and soil in order to minimize their adverse impacts on human health and the environment	Women are under-represented in decision-making related to management of chemicals in the life cycle.	

**Target 12.5**	By 2030, substantially reduce waste generation through prevention, reduction, recycling and reuse	Women are known to be more in contact with packaging, and their role in reduction, recycling and reuse of plastic waste in specific should be considered crucial.	
**Target 12.6**	Encourage companies, especially large and transnational companies, to adopt sustainable practices and to integrate sustainability information into their reporting cycle	Women are under-represented in leadership of large and transnational companies due to difficulties found in work-life imbalance in many countries.	
**Target 12.8**	By 2030, ensure that people everywhere have the relevant information and awareness for sustainable development and lifestyles in harmony with nature	Women in general are more interested in increasing awareness for sustainable development and lifestyles in harmony in nature, and therefore their role in achieving this target is crucial.	
**SDG 13 Take urgent action to combat climate change and its impacts**			

**Target 13.1**	By 2030, strengthen resilience and adaptive capacity to climate-related hazards and natural disasters worldwide	Climate change is experienced differently by women and men because of the interplay between gender, poverty and participation in the political arena. When taking action, the interplay between gender relations and the vulnerability of women needs to be taken into consideration. An understanding of gender relations is important for the transformation in social behaviour with climate change.	Herbert et al., 2020<br>Pearse, 2017<br>Equal Measures 2030, 2021
**Target 13.2**	By 2030, integrate climate change measures into national policies, strategies and planning	Since women are underrepresented in government agencies and policy-making, they are negatively affected by this. This also limits their agency in climate advocacy and mitigation measures.	
**Target 13.3**	By 2030, improve education, awareness-raising and human and institutional capacity on climate change mitigation, adaptation, impact reduction and early warning	Gender inequalities are evident, in that women, especially those living in the global South, are more prone to resource scarcity, poverty and risks, such as environmental stress, posed by climate change. Also, socio-cultural and economic circumstances negatively affect women's economic status (poverty) and education, and women become more susceptible to climate risks.	
**SDG 14 Conserve and sustainably use the oceans, seas and marine resources for sustainable development**			

**Target 14.1**	By 2025, prevent and significantly reduce marine pollution of all kinds, in particular from land-based activities, including marine debris and nutrient pollution	Women empowerment and education related to marine pollution in sea-based livelihoods is important so that they can lead sustainable development initiatives for pollution prevention	UN Women, 2018b<br>IISD, 2017<br>UN Sustainable Development Goals, 2020
**Target 14.2**	By 2020, sustainably manage and protect marine and coastal ecosystems to avoid significant adverse impacts, including by strengthening their resilience, and take action for their restoration in order to achieve healthy and productive oceans	Women are under-represented in policy and management in the fishing industry, so it is important to increase their participation in leadership and decision–making processes for the sustainable management of coastal ecosystems	
**Target 14.4**	By 2020, effectively regulate harvesting and end overfishing, illegal, unreported and unregulated fishing and destructive fishing practices and implement science-based management plans, in order to restore fish stocks in the shortest time feasible, at least to levels that can produce maximum sustainable yield as determined by their biological characteristics	Women are mostly affected by unsustainable fishing because the majority of them deal with fishing activities near shores, not having the resources for sophisticated equipment. Usually they possess only simple instruments for fishing.	

**Target 14.5**	By 2020, conserve at least 10 per cent of coastal and marine areas, consistent with national and international law and based on the best available scientific information	Equal participation and co-management of resources is important in order for policies of conservation to be accepted by coastal female communities. Also, equal access to knowledge production and information related to coastal conservation.	
**Target 14.7**	By 2030, increase the economic benefits to Small Island developing States and least developed countries from the sustainable use of marine resources, including through sustainable management of fisheries, aquaculture and tourism	Providing women in small islands developing states and least developed countries with equal opportunities in training and new skills acquisition in the fisheries sector can ensure a more equal access to resources and improve their economic conditions, thus contributing to the GDP increase by sustainable fisheries.	
**Target 14.a**	Increase scientific knowledge, develop research capacity and transfer marine technology, taking into account the Intergovernmental Oceanographic Commission Criteria and Guidelines on the Transfer of Marine Technology, in order to improve ocean health and to enhance the contribution of marine biodiversity to the development of developing countries, in particular small	Women are under-represented in marine science, so it is important to provide budget and support and to create a safe environment for them, so that their participation in science and the knowledge exchange in the field of marine technology and ocean-related industries can be increased.	

	island developing States and least developed countries		
**Target 14.b**	Provide access for small-scale artisanal fishers to marine resources and markets	Promotion of female fishing leaders and protecting their rights is important to increase their access to small-scale fisheries and marine resources markets, because often they are underpaid or without contracts or health and safety insurance, even though, according to the Food and Agriculture Organization, '*women represent nearly half of the estimated 180 million people worldwide working in fisheries and aquaculture'*.	
**Target 14.c**	Enhance the conservation and sustainable use of oceans and their resources by implementing international law as reflected in UNCLOS, which provides the legal framework for the conservation and sustainable use of oceans and their resources, as recalled in paragraph 158 of The Future We Want	Increased female representation in international advocacy can support the processes of ratification by countries of institutional frameworks, ocean-related instruments, etc., for conservation and sustainable use of the oceans and their resources.	
**SDG 15 Protect, restore and promote sustainable use of terrestrial ecosystems, sustainably manage forests, combat desertification, and halt and reverse land degradation and halt biodiversity loss**			
**Target 15.1**	By 2020, ensure the conservation, restoration and	Gender differentiated responsibilities vary region to region, but in many communities around the world,	Agu & Gore, 2020

	sustainable use of terrestrial and inland freshwater ecosystems and their services, in particular forests, wetlands, mountains and drylands, in line with obligations under international agreements	women act as primary caretakers and natural resource managers – procuring water and firewood, managing waste and providing health care, often through plant-based medicines. Women's roles also mean they hold vast knowledge on sources of water, storing and caring for seeds, and the diverse uses and benefits of plants, including for food, medicine, art, and avoiding and mitigating land degradation. This knowledge is crucial to preserving biodiversity, addressing climate change and supporting livelihoods.	Broeckhoven, 2014
**Target 15.3**	By 2030, combat desertification, restore degraded land and soil, including land affected by desertification, drought and floods, and strive to achieve a land degradation-neutral world	The role of women in international law and policy is still low, especially in developing countries, and the decision-making about combating desertification and restoration of land and soil is limited.	
**Target 15.5**	Take urgent and significant action to reduce the degradation of natural habitats, halt the loss of biodiversity and, by 2020, protect and prevent the extinction of threatened species	Especially in developing countries, the role of women in the reduction of natural habitat destruction and biodiversity loss has been considered important. Women need to be equally and actively involved in processes to conserve and sustainably use biodiversity because they play critical roles as primary land managers and resource users, and they face disproportionate impacts both from biodiversity loss and gender-blind conservation measures.	

**Target 15.8**	Take urgent action to end poaching and trafficking of protected species of flora and fauna and address both demand and supply of illegal wildlife products	Efforts to overcome challenges to combatting wildlife trafficking will likely benefit from increased attention to, and mainstreaming of, the role of women in wildlife trafficking, especially in Africa. The role of women has been considered low in Asia.	
**SDG 16 Promote peaceful and inclusive societies for sustainable development, provide access to justice for all and build effective, accountable and inclusive institutions at all levels**			
**Target 16.1**	Significantly reduce all forms of violence and related death rates everywhere	Significantly reduce all forms of violence and related death rates across all countries by 2030	Leal Filho et al., 2021<br><br>Institute for Economics and Peace, 2014<br><br>Pathfinders for Peaceful, Just and Inclusive Societies, 2017<br><br>UNICEF Office of Research, 2017<br><br>Bolaji-Adio, 2015<br><br>Adedayo Bolaji-Adio,

			Shakti, 2017
**Target 16.2**	End abuse, exploitation, trafficking and all forms of violence against and torture of children	Proportion of children aged 1–17 years who experienced any physical punishment and/or psychological aggression by caregivers in the past month.	Council of Europe, 2017<br><br>Hyder and Malik, 2007<br><br>Raman et al., 2020<br><br>UNICEF, 2009<br><br>Peterman & O'Donnell, 2020<br><br>World Health Organization, 2020<br><br>Save The Children International Asia, 2020<br><br>Fabbri et al., 2020
**Target 16.3**	Promote the rule of law at the national and international levels and ensure equal access to justice for all	Proportion of victims of violence in the previous 12 months who reported their victimisation to competent authorities	Hillis et al., 2016<br><br>Rabinovych, 2020<br>Beqiraj & McNamara, 2016<br>Quinn & Sannerholm, 2019<br>Government of Canada, 2020

			Lima & Gomez, 2019<br><br>Alffram, 2011<br><br>Open Government Partnership, 2019<br><br>Satterthwaite & Dhital, 2019<br><br>UNICEF & Regional Office for CEE/CIS, 2015 OECD, 2019<br><br>Manuel and Manuel, 2018
**Target 16.4**	By 2030, significantly reduce illicit financial and arms flows, strengthen the recovery and return of stolen assets and combat all forms of organized crime	Total value of inward and outward illicit financial flows	Arib, 2017<br><br>Reuter, 2017<br><br>Bromley et al., 2019<br><br>UNCTAD & UNODC, 2021<br><br>Fisher, 2020
**Target 16.5**	Substantially reduce corruption and bribery in all their forms	Substantially reduce corruption and bribery in all their forms across all countries by 2030.	Hoffiani, 2019<br><br>Whaites, 2016<br><br>Hope, 2020

			Mugellini and Villeneuve, 2019<br><br>V-Dem Institute, 2017<br><br>Department for International Development, 2015<br><br>Bahoo et al., 2020<br>Mackey et al., 2016<br><br>Sartor & Beamish, 2020
**Target 16.6**	Develop effective, accountable and transparent institutions at all levels	Primary government expenditures as a proportion of original approved budget, by sector (or by budget codes or similar)	United Nations, 2015<br>Tully, 2015<br><br>Poisson, 2016<br>OECD, 2019b<br>V-Dem Institute, 2015<br>Carothers and Brechenmacher, 2014<br><br>Blind, 2019<br><br>International Bank for Reconstruction and Development & World Bank, 2020

**Target 16.7**	Ensure responsive, inclusive, participatory and representative decision-making at all levels	Proportions of positions (by sex, age, persons with disabilities and population groups) in public institutions (national and Local legislatures, public service, and judiciary) compared to national distributions	Hayes & Bulat, 2017<br>World Health Organization, 2011<br>European Union Agency for Fundamental Rights, 2020<br>Mijatović, 2018
**Target 16.8**	Broaden and strengthen the participation of developing countries in the institutions of global governance	Proportion of members and voting rights of developing countries in international organisations	Glass & Newig, 2019<br>Qoraboyev, 2021<br>Weiss and Wilkinso, 2018<br>Gellers, 2016<br>OECD, 2019a
**Target 16.9**	By 2030, provide legal identity for all, including birth registration	Proportion of members and voting rights of developing countries in international organisations	van der Straaten, 2015<br>Manby, 2017<br>Vandenabeele & Lao, 2007

			Dahan & Gelb, 2015<br>Perrault & Arellano, 2011
**Target 16.10**	Ensure public access to information and protect fundamental freedoms, in accordance with national legislation and international agreements	Number of verified cases of killing, kidnapping, enforced disappearance, arbitrary detention and torture of journalists, associated media personnel, trade unionists and human rights advocates in the previous 12 months	Slutskiy, 2020<br>Berger, 2020<br>UNESCO, 2018b<br>Phogat, 2015<br>United Nations, 2008
**Target 16.11**	Strengthen relevant national institutions, including through international cooperation, for building capacity at all levels, in particular in developing countries, to prevent violence and combat terrorism and crime	Existence of independent national human rights institutions in compliance with the Paris Principles	Manuel & Manuel, 2018<br>Hilderbrand, 2015<br>Zamfir, 2020
**Target 16.12**	Promote and enforce non-discriminatory laws and policies for sustainable development	Proportion of population reporting to having personally felt discriminated against or harassed in the previous 12 months on the basis of a ground of discrimination prohibited under international human rights law	Pisano et al., 2015<br>British Council, 2018<br>International Organization for Migration & Joint Migration and

			Development Initiative, 2015<br><br>Government of Andhra Pradesh, 2017<br><br>United Nations Human Rights, 2020<br><br>Lindsey & Chapman, 2017<br><br>United Nations System Chief Executives Board for Coordination, 2017
**SDG 17 Strengthen the means of implementation and revitalize the global partnership for sustainable development**			
**Target 17.1**	Strengthen domestic resource mobilization, including through international support to developing countries, to improve domestic capacity for tax and other revenue collection	Creating equal working conditions and opportunities for women especially in the Global South, where the majority of women do not work formally, and thus do not contribute to the income taxes.	Joshi et al., 2020<br><br>UN Women, 2020b<br><br>OECD, 2020
**Target 17.3**	Mobilize additional financial resources for developing countries from multiple sources	It is important that foreign aid considers gender equity and provides funds for empowering woman. According to UN Women, ‘*Only 5 per cent of foreign aid funds*	

		had gender equality as a principle objective in 2012-2013'.	
**Target 17.7**	Promote the development, transfer, dissemination and diffusion of environmentally sound technologies to developing countries on favourable terms, including on concessional and preferential terms, as mutually agreed	Clean technologies related to everyday work and activities of women (i.e., clean cooking technologies) can affect their health and improve their life conditions.	
**Target 17.8**	Fully operationalize the technology bank and science, technology and innovation capacity-building mechanism for least developed countries by 2017 and enhance the use of enabling technology, in particular information and communications technology	Women are under-represented in IT jobs, top management and academic careers, but it is important that woman are included in digital transformation and that there is no digital gender divide. In many countries, women have less access to technological devices. According to the OECD, approx. 327 million fewer women than men in the world can use a smartphone or can have access to mobile internet.	
**Target 17.10**	Promote a universal, rules-based, open, non-discriminatory and equitable multilateral trading system under the World Trade Organization, including through the conclusion of	Women can be empowered and protected by fair trade practices, which consider labour conditions, prevent discrimination, promote equal access to employment, etc.	

	negotiations under its Doha Development Agenda		
**Target 17.12**	Realize timely implementation of duty-free and quota-free market access on a lasting basis for all least developed countries, consistent with World Trade Organization decisions, including by ensuring that preferential rules of origin applicable to imports from least developed countries are transparent and simple, and contribute to facilitating market access		
**Target 17.17**	Encourage and promote effective public, public-private and civil society partnerships, building on the experience and resourcing strategies of partnerships	It is important that female organised networks or civil society organisations that deal with gender equality, women rights, etc. are part of the partnerships and coalitions for goals, in order to increase their representation in national and international processes	
**Target 17.19**	By 2030, build on existing initiatives to develop measurements of progress on sustainable development that complement gross domestic product, and support statistical	Many countries do not have enough data that identify women's issues or gender-based data, which is a critical aspect for supporting policies for gender equality. According to UN Women, around a third of countries have an office for gender statistics.	

	capacity-building in developing countries		

References

Abbas, K., Li, S., Xu, D., Baz, K., & Rakhmetova, A. (2020). Do socioeconomic factors determine household multidimensional energy poverty? Empirical evidence from South Asia, *Energy Policy, 146,* 111754. https://doi.org/10.1016/j.enpol.2020.111754

Abdulkadir, I., Kumar, J. S., & Noon, M. (2019). Ratio of Land Consumption Rate to the Population Growth Rate- A Case of Metropolitan Gombe. 2019120047. https://doi.org/10.20944/preprints201912.0047.v1

Abebe, O. J. (2016). An Analysis of Women and Sustainable Development Goals. UN Women. http://dx.doi.org/10.2139/ssrn.2797855

Alffram, H. (2011). Equal Access to Justice A Mapping of Experiences. Sida. https://publikationer.sida.se/contentassets/8d1d0ea3d9464589af9259c07937ce35/equal-access-to-justice-a-mapping-of-experiences_3124.pdf

Agu, H., & Gore, M. L. (2020). Women in wildlife trafficking in Africa: A synthesis of literature. *Global ecology and Conservation*, *23*. https://www.iied.org/role-influence-impact-women-biodiversity-conservation

Agyemang, F. S. K., & Morrison, N. (2017). Recognising the barriers to securing affordable housing through the land use planning system in Sub-Saharan Africa: A perspective from Ghana. *Urban Studies, 55*(12), 2640-2659. https://doi.org/10.1177/0042098017724092

Alarcón, D. M., & Cole, S. (2019). No sustainability for tourism without gender equality. *Journal of Sustainable Tourism*, *27*(7), 903–919.

Arimah, B. C. (2001). *Slums as expressions of social exclusion: explaining the prevalence of slums in African countries.* https://www.oecd.org/dev/pgd/46837274.pdf

Arib, E. (2017). *Policy, SDGs and fighting corruption for the people*. Transparency International https://images.transparencycdn.org/images/2018_Report_PolicySDGsandFightingCorruption_EN.pdf

Bahoo, S., B., Ilan, A., & Paltrinieri, A. (2020). Corruption in international business: A review and research agenda. *International Business Review*, *29*(4), 101660. https://doi.org/10.1016/j.ibusrev.2019.101660

Beqiraj, J., & McNamara, L. (2016). Children and Access to Justice in the Agenda for Sustainable Development. Briefing Paper by the Bingham Centre for the Rule of Law. https://www.biicl.org/documents/1022_children_and_access_to_justice_report_may_2016_full.pdf?showdocument=1

Berger, G. (2020). New Opportunities in Monitoring Safety of Journalists through the UN's 2030 Sustainable Development Agenda. *Media and Communication, 8*(1). http://dx.doi.org/10.17645/mac.v8i1.2660

Bhandari, M. P., & and Shvindina, H. (2019). *Reducing Inequalities Towards Sustainable Development Goals: Multilevel Approach*. River Publishers, Denmark/the Netherlands.

Blei, A. M., Angel, S., Civco, D. L., Liu, Y., & Zhang, X. (2018). Accuracy Assessment and Map Comparisons for Monitoring Urban Expansion: The Atlas of Urban Expansion and the Global Human Settlement. Lincoln Institute of Land Policy.

Blind, P. K. (2019). How relevant is governance to financing for development and partnerships? Working Paper No. 162 ST/ESA/2019/DWP/162. United Nations Department of Economic and Social Affairs. https://www.un.org/esa/desa/papers/2019/wp162_2019.pdf

Bolaji-Adio, A. (2015). The Challenge of Measuring SDG 16: What Role for African Regional Frameworks? Discussion Paper No. 175. https://ecdpm.org/wp-content/uploads/DP175-Challenge-Measuring-SDG16-May-2015.pdf

British Council (2018). *A cultural relations contribution to peace, justice and strong institutions.* https://www.britishcouncil.org/sites/default/files/j082_sustainable_development_goals_report_final_web.pdf

Broeckhoven, N. (2014). Biodiversity Loss and Climate Change: Gender Issues in International Law and Policy. *DiGeSt. Journal of Diversity and Gender Studies*, *1*(2), 23-38.

Bromley, M., Caparini, M., & Malaret, A. (2019). Measuring illicit arms and financial flows: improving the assessment of sustainable development goal 16, SIPRI Background Paper. https://www.sipri.org/sites/default/files/2019-07/bp_1907_sdg_16.pdf

Buhmann, K., Jonsson, J., & Fisker, M. (2019). Do no harm and do more good too: connecting the SDGs with business and human rights and political CSR theory. *Corporate Governance*, *19*(3), 389-403.

Campbell, B. M., Hansen, J., Rioux, J., Stirling, C. M., Twomlow, S., & Wollenberg, E. L. (2018). Urgent action to combat climate change and its impacts (SDG 13): transforming agriculture and food systems. *Current Opinion in Environmental Sustainability*, *34*, 13-20. https://doi.org/10.1016/j.cosust.2018.06.005

Carothers, T., & Brechenmacher, S. (2014). Accountability, transparency, participation, and inclusion. A New Development Consensus? Carnegie Endowment for International Peace.

Chattopadhyay, A., Mukherjee, A., Sudha, G. (2016). Prevailing Basic Facilities in Slums of Greater Mumbai. https://www.iipsindia.ac.in/sites/default/files/IIPS_Working_Paper_No13.pdf

Cho, Y., Park, J., & Park, H. Y. (2018). Women Leaders in the Corporate Sector. In Y. Cho & G.N. McLean (Eds.), *Korean Women in Leadership, Current Perspectives on Asian Women in Leadership* (pp. 121-139). Palgrave Macmillan, Cham.

Clos, J. (2016). *A New Urban Agenda for the 21st century: The role of urbanization in sustainable development*. OECD Regional Outlook 2016.

Cohen, B. (2006). Urbanization in Developing Countries: Current Trends, Future Projections, and Key Challenges for Sustainability. *Technology in Society*, *28*(1-2), 63-80. https://doi.org/10.1016/j.techsoc.2005.10.005

Council of Europe (2017). Ending all forms of violence against children by 2030: The Council of Europe's contribution to the 2030 Agenda and the Sustainable Development Goals.

https://violenceagainstchildren.un.org/sites/violenceagainstchildren.un.org/files/2030_agenda/sdg_leaflet.pdf.pdf

Dahan, M., & Gelb, A. (2015). The role of identification in the post-2015 development agenda. World Bank Working Paper. http://pubdocs.worldbank.org/en/149911436913670164/World-Bank-Working-Paper-Center-for-Global-Development-Dahan-Gelb-July2015.pdf

Damodaran, A., Jörgensen, K., Schreurs, M., Beermann, J., & Ollier, L. (2015). Sustainable cities – inclusive, green and competitive. https://smartnet.niua.org/sites/default/files/resources/giz20162d0389en2dindo2dgerman2dsustainable2dcities.pdf

Daniel, K. (2016). *Public Spaces. A key tool to achieve the sustainable development goals.* https://healthbridge.ca/images/uploads/library/Final_Electronic.pdf

Department for International Development (2015). Why corruption matters: understanding causes, effects and how to address them Evidence paper on corruption. Department for International Development, UK Government. https://assets.publishing.service.gov.uk/government/uploads/system/uploads/attachment_data/file/406346/corruption-evidence-paper-why-corruption-matters.pdf

Desai, D. (2020). Urban Densities and the Covid-19 Pandemic: Upending the Sustainability Myth of Global Megacities. Observer research foundation. https://www.orfonline.org/wp-content/uploads/2020/05/ORF_OccasionalPaper_244_PandemicUrbanDensities.pdf

Devisscher, T., Konijnendijk, C., Nesbitt, L., Lenhart, J., Salbitano, F., Cheng, Z. C., Lwasa, S., & van den Bosch, M. (2019). SDG 11: Sustainable Cities and Communities – Impacts on Forests and Forest-Based Livelihoods. In P. Katila, C. J. Pierce Colfer, W. de Jong, P. Pacheco, & G. Winkel, G. (Eds.), *Sustainable Development Goals: Their Impacts on Forests and People* (pp 349-385), Cambridge University Press.

DIAUD/ CBM (2016). The Inclusion Imperative: Towards Disability-inclusive and Accessible Urban Development Key Recommendations for an Inclusive Urban Agenda https://www.cbm.org/fileadmin/user_upload/Publications/The-Inclusion-Imperative-Towards-Disability-Inclusive-and-Accessible-Urb....pdf

Dugarova, E. (2018). Gender equality as an accelerator for achieving the Sustainable Development Goals. Discussion Paper. United Nations Entity for Gender Equality and the Empowerment of Women, New York, USA.

Equal Measures 2030 (2021). *Why SDG 13 matters for gender equality*. https://data.em2030.org/goals/sdg13/

ESCAP (2017). Transport and Communications Bulletin for Asia and the Pacific No. 87 Transport and Sustainable Development Goals. https://www.unescap.org/sites/default/files/publications/bulletin87_Fulltext.pdf

European Environment Agency (2015). Urban sustainability issues — What is a resource-efficient city? EEA Technical report No 23/2015 https://www.eea.europa.eu/publications/resource-efficient-cities/file

European Institute for Gender Equality (2020). Care. Gender sensitive infrastructure. https://op.europa.eu/en/publication-detail/-/publication/4bec6067-cfd4-11ea-adf7-01aa75ed71a1

European Union Agency for Fundamental Rights (2020). Strong and effective national human rights institutions challenges, promising practices and opportunities. https://fra.europa.eu/sites/default/files/fra_uploads/fra-2020-strong-effective-nhris_en.pdf

Fabbri, C., Bhatia, A., Petzold, M., Jugder, M., Guedes, A., Cappa, C., & Devries, K. (2020). The right to protection ending violence against children. *Child Abuse & Neglect*, 104897 https://doi.org/10.1016/j.chiabu.2020.104897

Fanzo, J. (2019). Healthy and Sustainable Diets and Food Systems: the Key to Achieving Sustainable Development Goal 2?. *Food ethics*, *4*, 159–174. https://doi.org/10.1007/s41055-019-00052-6

Fisher, P. G. (2020). *Making the Financial System Sustainable*. Cambridge University Press. https://doi.org/10.1017/9781108908269

Fontefrancesco M. F. (2019). Food Commodity Market: History and Impact of Food Trading Toward SDG2. In W. Leal Filho, A. M. Azul, L. Brandli, P. G. Özuyar, & T. Wall (Eds.), *Zero Hunger. Encyclopedia of the UN Sustainable Development Goals*. Springer, Cham. https://doi.org/10.1007/978-3-319-69626-3_13-1

Franco, I. B., & Minnery, J. (2020). SDG 1 No Poverty. In I. Franco, T. Chatterji, E. Derbyshire, & J. Tracey (Eds.), *Actioning the Global Goals for Local Impact. Science for Sustainable Societies*. Springer, Singapore. https://doi.org/10.1007/978-981-32-9927-6_2

Freistein, K., & Mahlert, B. (2015). The Role of Inequality in the Sustainable Development Goals, Conference Paper, University of Duisburg-Essen.

Gellers, J. C. (2016). Crowdsourcing global governance: sustainable development goals, civil society, and the pursuit of democratic legitimacy. *International Environmental Agreements, 16*, 415–432. https://doi.org/10.1007/s10784-016-9322-0

Ghosh-Jerath, S., Kapoor, R., Singh, A., Downs, S., Barman, S., & Fanzo. J. (2020). Leveraging Traditional Ecological Knowledge and Access to Nutrient-Rich Indigenous Foods to Help Achieve SDG 2: An Analysis of the Indigenous Foods of Sauria Paharias, a Vulnerable Tribal Community in Jharkhand, India. *Frontiers in Nutrition, 7*(61). https://doi.org/10.3389/fnut.2020.00061

Glass, L-M., & Newig, J. (2019). Governance for achieving the Sustainable Development Goals: How important are participation, policy coherence, reflexivity, adaptation and democratic institutions? *Earth System Governance, 2,* 100031. https://doi.org/10.1016/j.esg.2019.100031

Gopalan, K., & Venkataraman, M. (2015). Affordable housing: Policy and practice in India. *IIMB Management Review*, *27*(2), 129-140.

Government of Andhra Pradesh (2017). Achieving sustainable development goals 2030 baseline, targets and strategy. http://4dj7dt2ychlw3310xlowzop2.wpengine.netdna-cdn.com/wp-content/uploads/2017/07/Andhra-Pradesh_Vision-2029.pdf

Government of Canada (2020). *An Open Justice Commitment for Canada - Discussion Paper. Government of Canada, Department of Justice*. https://www.justice.gc.ca/eng/rp-pr/other-autre/trans/open-ouvert/ojcdp-eejodt.html

Grogan, J. (2020). America's legacy cities: building an equitable renaissance. Policy brief. Lincoln Institute of Land Policy.

Guedes Vidal, D., Barros, N., & Leandro Maia, R. (2019). Public and Green Spaces in the Context of Sustainable Development. In W. Leal Filho, A. M. Azul, L. Brandli, P. G. Özuyar, & T. Wall (Eds.), *Sustainable Cities and Communities Living Edition*. https://doi.org/10.1007/978-3-319-71061-7_79-1

Hayes, A. M., & Bulat, J. (2017). Disabilities Inclusive Education Systems and Policies Guide for Low- and Middle-Income Countries. RTI Press Publication No. OP-0043-1707. Research Triangle Park, NC: RTI Press. https://doi.org/10.3768/rtipress.2017.op.0043.1707

Hansen, N., Huis, M. A., & Lensink, R. (2020). Microfinance Services and Women's Empowerment. In L. San-Josem, J. Retolaza, & L. van Liedekerke, L. (Eds.), *Handbook on Ethics in Finance. International Handbooks in Business Ethics*. Springer, Cham. https://doi.org/10.1007/978-3-030-00001-1_4-1

Herbert, R., Falk-Kresinski, H. J., & Plume, A (2020). Sustainability through a gender lens: The extent to which researh on UN sustainable development goals (SDGs) includes sex and gender consideration. http://dx.doi.org/10.2139/ssrn.3689205

Hilderbrand, M. (2015). Benefits and Costs of the Governance & Institutions Targets for the Post2015 Development Agenda Post-2015 Consensus. Copenhagen Consensus Center. https://www.copenhagenconsensus.com/sites/default/files/governance_assessment_-_hilderbrand.pdf

Hillis, S., Mercy, J., Amobi, A., & Kress, H. (2016). Global Prevalence of Past-year Violence Against Children: A Systematic Review and Minimum Estimates. *Pediatrics*, 2015-4079. https://doi.org/10.1542/peds.2015-4079

Hoek-Smit, M., Kyung-Hwan, K., & Wachter, S. (2020). Cities with Affordable Housing: Fulfilling the New Urban Agenda. https://realestate.wharton.upenn.edu/wp-content/uploads/2020/06/Working-paper-829.pdf

Hoffiani, M. (2019). The Nexus between Corruption, Sustainable Development and Rule of Law. https://www.diva-portal.org/smash/get/diva2:1352722/FULLTEXT01.pdf

Hoornweg, D., & Pope, K. (2017). Population predictions for the world's largest cities in the 21st century. *Environment & Urbanization, International Institute for Environment and Development (IIED), 29*(1), 195–216. http://dx.doi.org/10.1177/0956247816663557

Hope, K. R. (2020). Corruption Reduction as a Target of the Sustainable Development Goals: Applying Indicators and Policy Frameworks. In J. Blaustein, K. Fitz-Gibbon, N. W. Pino, & R. White (Eds.), *The Emerald Handbook of Crime, Justice and Sustainable Development* (pp. 105-130), Emerald Publishing Limited.

Hyder, A. A., & Malik, A. M. (2007). Violence against Children: A Challenge for Public Health in Pakistan. *Journal of Health, Population and Nutrition*, *25*(2),168-178. https://www.ncbi.nlm.nih.gov/pmc/articles/PMC2753994/pdf/jhpn0025-0168.pdf

Idowu, S. O., Schmidpeter, R., & Zu, L. (Eds.). (2020). *The Future of the UN Sustainable Development Goals: Business Perspectives for Global Development in 2030*. Springer International Publishing. http://dx.doi.org/10.1007/978-3-030-21154-7

Institute for Economics and Peace (2014). Measuring goal 16 identifying priority indicators based on key statistical and normative criteria. https://www.economicsandpeace.org/wp-content/uploads/2015/06/Measuring-Goal-16.pdf

International Bank for Reconstruction and Development/World Bank (2020). Building effective, accountable, and inclusive institutions in Europe and Central Asia. https://www.pefa.org/sites/pefa/files/resources/downloads/Building-Effective-Accountable-and-Inclusive-Institutions-in-Europe-and-Central-Asia-Lessons-from-the-Region.pdf

International Council for Science (2011). *Report of the ICSU Planning Group on Health and Wellbeing in the Changing Urban Environment: a Systems Analysis Approach*. International Council for Science, Paris. https://sph.umd.edu/sites/default/files/files/health-and-wellbeing-in-the-changing-urban-environment.pdf

International Labour Organisation (2012). Text of the Recommendation concerning National Floors of Social Protection. International Labour Convention. 14A ILO.

International Organization for Migration and Joint Migration and Development Initiative (2015). White Paper mainstreaming migration into local development planning and beyond. International Organization for Migration, United Nations Development Programme. https://publications.iom.int/system/files/pdf/whitepaper_mainstreaming.pdf

Iqbal, N., Gkiouleka, A., Milner, A., Montag, D., & Gallo, V. (2018). Girls' hidden penalty: analysis of gender inequality in child mortality with data from 195 countries. *BMJ Global Health*, *3*(5), e001028. https://doi.org/10.1136/bmjgh-2018-001028

IISD (2017). *Achieve Gender Equality to Deliver the SDGs*. http://sdg.iisd.org/commentary/policy-briefs/achieve-gender-equality-to-deliver-the-sdgs/

JLL (2016). Affordable Housing in India. Key Initiatives for Inclusive Housing for All https://smartnet.niua.org/sites/default/files/resources/Affordable%20Housing-ICC%20-%20Final.pdf

Joshi, A., Kangave, J., & Boogaard, V. (2020). Gender and Tax Policies in the Global South. https://opendocs.ids.ac.uk/opendocs/bitstream/handle/20.500.12413/15450/817_Gender_and_Tax.pdf

Kaltenborn, M. (2017). Overcoming Extreme Poverty by Social Protection Floors – Approaches to Closing the Right to Social Security Gap. *Law and Development Review*, *10*(2). https://doi.org/10.1515/ldr-2017-0014

Katila, P., McDermott, C., Larson, A., Aggarwal, S., & Giessen, l. (2020). Forest tenure and the Sustainable Development Goals – A critical view. *Forest Policy and Economics*, *120*, 102294. https://doi.org/10.1016/j.forpol.2020.102294

Kaur, J. (2018). Impact Assessment of Access to Basic Services for Urban Poor in Chandigarh City, India. *Asian Journal of Public Affairs*, *11*(1), e2, Lee Kuan Yew School of Public Policy Research Paper No. 18-14. https://papers.ssrn.com/sol3/papers.cfm?abstract_id=3258884

Keivani, R. (2010). A review of the main challenges to urban sustainability. *International Journal of Urban Sustainable Development*, *1*(1-2), 5-16. https://doi.org/10.1080/19463131003704213

Kett, M., Cole, E., & Turner, J. (2020) Disability, Mobility and Transport in Low- and Middle-Income Countries: A Thematic Review. *Sustainability*, *12*(2), 589. https://doi.org/10.3390/su12020589

Klarin, T. (2018). The Concept of Sustainable Development: From its Beginning to the Contemporary Issues. *Zagreb International Review of Economics and Business*, *21*(1), 67-94. https://doi.org/10.2478/zireb-2018-0005

Konte, M. (2020). Female Policymakers and Women's Well-Being in Africa. In M. Konte,, & N. Tirivayi (Eds.), *Women and Sustainable Human Development. Gender, Development and Social Change.* Palgrave Macmillan, Cham. https://doi.org/10.1007/978-3-030-14935-2_18

Lang, V. F., & Lingnau, H. (2015). Defining and Measuring Poverty and Inequality Post-2015. https://doi.org/10.1002/jid.3084

Larson, P. D., & Larson, N. M. (2019). The Hunger of Nations: An Empirical Study of Inter-relationships among the Sustainable Development Goals (SDGs). *Journal of Sustainable Development*; *12*(6). https://doi.org/10.5539/jsd.v12n6p39

Leach, M. (Ed.). (2015). *Gender Equality and Sustainable Development*. Routledge, 1st edition. https://www.routledge.com/Gender-Equality-and-Sustainable-Development/Leach/p/book/9781138921313

Leal Filho, W., Azul, A. M., Brandli, L., Salvia, A. L., Özuyar, P. G., & Wall, T. (Eds.). (2021). *Peace, Justice and Strong Institutions*. Springer, Cham https://doi.org/10.1007/978-3-319-71066-2

Lima, V., & Gomez, M. (2019). Access to Justice: Promoting the Legal System as a Human Right. In W. Leal Filho, A. M. Azul, L. Brandli, A. L. Salvia, P. G. Özuyar, & T. Wall (Eds.), *Peace, Justice and Strong Institutions Living*. Springer Publishing. https://doi.org/10.1007/978-3-319-71066-2_1-1

Lindsey, I., & Chapman, T. (2017). Enhancing the Contribution of Sport to the Sustainable Development Goals. Commonwealth Secretariat. https://www.sportanddev.org/sites/default/files/downloads/enhancing_the_contribution_of_sport_to_the_sustainable_development_goals_.pdf

Liu, Z., de Jong, M., Hertogh, M., & Dong, L. (2020). Towards inclusive urban accessibility: framework and methodology for urban transport inclusiveness assessment - The Case of Xiong'an New Area. The 8th World Sustainability Forum.

Lucci, P., Bhatkal, T., Khan, A., & Berliner, T. (2015). What works in improving the living conditions of slum dwellers A review of the evidence across four programmes. https://www.odi.org/sites/odi.org.uk/files/odi-assets/publications-opinion-files/10188.pdf

Mackey, T. K., Kohler, J. C., Savedoff, W. D., Vogl, F., Lewis, M., Sale, J., Michaud, J., & Vian, T. (2016). The disease of corruption: views on how to fight corruption to advance 21st century global health goals. *BMC Med*, *14*, 149. https://doi.org/10.1186/s12916-016-0696-1

Manandhar, M., Hawkes, S., Buse, K., Nosrati, E., & Magar, V. (2018). Gender, health and the 2030 agenda for sustainable development. *Bulletin World Health Organization, 96*(9), 644–653. https://doi.org/10.2471/BLT.18.211607

Manby, B. (2017). *Legal identity for all" and childhood statelessness. Institute on Statelessness and Inclusion*. http://children.worldsstateless.org/3/childhood-statelessness-and-the-sustainable-development-agenda/legal-identity-for-all-and-childhood-statelessness.html

Mansell, P., Philbin, S. P., & Broyd, T. (2020). Development of a New Business Model to Measure Organizational and Project-Level SDG Impact—Case Study of a Water Utility Company. *Sustainability, 12*(16), 6413. https://doi.org/10.3390/su12166413

Manuel, M., & Manuel, C. (2018). Achieving equal access to justice for all by 2030 Lessons from global funds. Working paper 537.https://www.odi.org/sites/odi.org.uk/files/resource-documents/12307.pdf

Mijatović, D. (2018). *Paris Principles at 25: Strong National Human Rights Institutions Needed More Than Ever.* https://www.coe.int/en/web/commissioner/-/paris-principles-at-25-strong-national-human-rights-institutions-needed-more-than-ever

Mugellini, G., & Villeneuve, J-P. (2019). Monitoring the Risk of Corruption at International Level: The Case of the United Nations Sustainable Development Goals. *European Journal of Risk Regulation, 10*(1), 201–207. https://doi.org/10.1017/err.2019.16

Nabutola, W. (2004). Affordable Housing in Kenya: A Case Study of Policy on Informal Settlements Kenya. 3rd FIG Regional Conference Jakarta, Indonesia, October 3-7, 2004. https://www.humanitarianlibrary.org/sites/default/files/2013/07/ts_01_2_nabutola.pdf

Nallathiga, R. (2019). Housing for the Urban Poor: The Case of Chandigarh Model. NAGARLOK VOL. LI, Part 2, 142-151.

Nicolau, R., David, J., Caetano, M., & Pereira, J. M. C. (2019). Ratio of Land Consumption Rate to Population Growth Rate—Analysis of Different Formulations Applied to Mainland Portugal. *International Journal of Geo-Information, 8*(1), 10. https://doi.org/10.3390/ijgi8010010

Norichika, K., |& Biermann, F. (Eds.). (2017). *Governing through Goals: Sustainable Development Goals as Governance Innovation*. Cambridge, MA: MIT Press https://www.earthsystemgovernance.org/publication/governing-through-goals-sustainable-development-goals-as-governance-innovation/

Nygård, H. M. (2017). Achieving the sustainable development agenda: The governance – conflict nexus. *International Area Studies Review*, *20*(1), 3–18.

Nzau, B., & Trillo, C. (2020). Affordable Housing Provision in Informal Settlements through Land Value Capture and Inclusionary Housing. *Sustainability*, *12*(15), 5975. https://doi.org/10.3390/su12155975

OECD (2018). Policy Coherence for Sustainable Development 2018 - Towards Sustainable and Resilient Societies. https://www.oecd-ilibrary.org/development/policy-coherence-for-sustainable-development-2018_9789264301061-en

OECD (2019a). Governance frameworks to ensure equal access to justice and citizens' legal empowerment. https://www.oecd-ilibrary.org/sites/cae781ce-en/index.html?itemId=/content/component/cae781ce-en

OECD (2019b). Governance as an SDG Accelerator Country Experiences and Tools https://www.oecd.org/publications/governance-as-an-sdg-accelerator-0666b085-en.htm

OECD (2020). Bridging the digital gender divide. http://www.oecd.org/digital/bridging-the-digital-gender-divide.pdf

Olinto, P., Beegle, K., Sobrado, C., & Uematsu, H. (2013). The State of the Poor: Where Are The Poor, Where Is Extreme Poverty Harder to End, and What Is the Current Profile of the World's Poor? The World Bank. http://gesd.free.fr/wbpoor13.pdf on 12.01.21

Open Government Partnership (2019). Access to Justice, Open Government Partnership Global Report Democracy beyond the ballot box. Justice Policy Series, Part I. https://www.opengovpartnership.org/wp-content/uploads/2019/09/Justice-Policy-Series-Access-to-Justice.pdf

Overseas Development Institute (2018). Migration and the 2030 Agenda for Sustainable Development. https://www.odi.org/sites/odi.org.uk/files/resource-documents/12422.pdf

Pandey, U.C., & Kumar, C. (2019). "The Relationship of SDG5 to Other Goals", SDG5 – Gender Equality and Empowerment of Women and Girls (Concise Guides to the United Nations Sustainable Development Goals) (pp. 103-120). Emerald Publishing Limited, Bingley. https://doi.org/10.1108/978-1-78973-521-520191008

Pathfinders for Peaceful, Just and Inclusive Societies (2017). The Roadmap for Peaceful, Just and Inclusive Societies – A Call to Action to Change our World. New York: Center on International Cooperation. https://cic.nyu.edu/sites/default/files/sdg16_roadmap_en_20sep17.pdf

Pearse, R. (2017). Gender and climate change. *WIREs Climate Change, 8*, e451. https://doi.org/10.1002/wcc.451

Perrault, N., & Arellano, B. (2011). A rights-based approach to birth registration in Latin America and the Caribbean. UNICEF – Regional Office for Latin America and the Caribbean Documentation Centre. https://repositorio.cepal.org/bitstream/handle/11362/35984/1/challenges-13-ECLAC-UNICEF_es.pdf

Peterman, A., & O'Donnell, M. (2020). COVID-19 and Violence against Women and Children A Second Research Round Up. https://resourcecentre.savethechildren.net/node/18275/pdf/covid-19-and-violence-against-women-and-children-second-research-round.pdf

Phogat, V. (2015). Right to information in consonance with right to privacy https://cic.gov.in/sites/default/files/Internship%20Research%20Paper-%20Vratika%20Phogat.pdf

Pisano, P., Lange, L., Berger, G., & Hametner, M. (2015). The Sustainable Development Goals (SDGs) and their impact on the European SD governance framework Preparing for the post-2015 agenda. European Sustainable Development Network. https://www.sd-network.eu/quarterly%20reports/report%20files/pdf/2015-January-The_Sustainable_Development_Goals_(SDGs)_and_their_impact_on_the_European_SD_governance_framework.pdf

Poisson, M. (2016). Promoting public access to information is key to improving transparency and accountability. http://www.iiep.unesco.org/en/promoting-public-access-information-key-improving-transparency-and-accountability

Pudaruth, S., Devi Juwaheer, T., & Seewoo, Y. D. (2015). Gender-based differences in understanding the purchasing patterns of eco-friendly cosmetics and beauty care products in Mauritius: a study of female customers. *Social Responsibility Journal, 11*(1), 179-198. https://doi.org/10.1108/SRJ-04-2013-0049

Qoraboyev, I. (2021). Global governance. In K. De Feyter, G. E. Turkeli, & S. De Moerloose, (Eds.), *Law and Development Encyclopedia* (pp. 99-103). Edward Elgar.

Queen Mary University of London (2018). *Gender inequality could be driving the deaths of girls under five. Science Daily*. www.sciencedaily.com/releases/2018/10/181030184504.htm

Quinn, S., & Sannerholm, S. (2019). Rule of law washing and the Sustainable Development Goals. ILAC Policy Brief 4. http://ilacnet.org/wp-content/uploads/2019/12/Using-SDG16-for-rule-of-law-washing_20191217_V4_Final2.pdf

Rabinovych, M. (2020). Where Economic Development Meets the Rule of Law? Promoting Sustainable Development Goals Through the European Neighborhood Policy. *Brill Open Law, 2*(1), 140–174. https://doi.org/10.1163/23527072-20191017

Rai, S. M., Brown, B. D., & Ruwanpura, K. N. (2019). SDG 8: Decent work and economic growth – A gendered analysis. *World Development*, *113*, 368-380. https://doi.org/10.1016/j.worlddev.2018.09.006

Raman, S., Muhammad, T., Goldhagen, J., Seth, R., Kadir, A., Bennett, S., D'Annunzio, D., Spencer, N..J., Bhutta, Z. A., & Gerbaka, B. (2020). Ending violence against children: What can global agencies do in partnership? *Child Abuse & Neglect*, 104733. https://doi.org/10.1016/j.chiabu.2020.104733

Reuter, P. (2017). Illicit Financial Flows and Governance: The Importance of Disaggregation. Background paper for World Development Report 2017. http://documents1.worldbank.org/curated/en/538841487847427218/pdf/112973-WP-PUBLIC-WDR17BPIllicitFinancialFlows.pdf

Rodríguez-Pose, A., & Hardy, D. (2015). Addressing poverty and inequality in the rural economy from a global perspective, *Applied Geography, 61*, 11-23. https://doi.org/10.1016/j.apgeog.2015.02.005

Ros-Tonen, M. A. F., Agergaard, J., & Zoomers, A. (2016). SDG Policy Brief #3 Support positive links between urban, peri-urban and rural areas. https://doi.org/10.13140/RG.2.1.1869.9761

Santiago Pineda, V., Meyers, S., & Cruz, J. P. (2017). The Inclusion Imperative. Forging an Inclusive New Urban Agenda. *The Journal of Public Space, 2*(4),1. https://doi.org/10.5204/jps.v2i4.138

Sartor, M.A., & Beamish, P.W. (2020). Private Sector Corruption, Public Sector Corruption and the Organizational Structure of Foreign Subsidiaries. *Journal of Business Ethics*, *167,* 725–744. https://doi.org/10.1007/s10551-019-04148-1

Santoro, P. F. (2015). Urban planning to provide affordable housing in infrastructured areas, with social cohesion, through the market: Real estate profitability or right to the city assurance? RC21 International Conference on "The Ideal City: between myth and reality. Representations, policies, contradictions and challenges for tomorrow's urban life" Urbino (Italy). http://www.rc21.org/en/conferences/urbino2015/.

Satterthwaite, D. (2017). Successful, safe and sustainable cities: towards a New Urban Agenda. *Commonwealth Journal of Local Governance*. https://doi.org/10.5130/cjlg.v0i19.5446

Satterthwaite, M. L., & Dhital, S. (2019). Measuring Access to Justice: Transformation and Technicality in SDG 16.3. *Global policy, 10*(S1), 96-109. https://doi.org/10.1111/1758-5899.12597

Save The Children International Asia (2020). Because we matter. Addressing COVID-19 and Violence Against Girls in Asia-Pacific. https://resourcecentre.savethechildren.net/node/17928/pdf/pi_stc_becausewematterpolicybrief-final.pdf

SDG Compass (2021). SDG 10: Reduce inequality within and among countries. https://sdgcompass.org/sdgs/sdg-10/

Seto, K. C., Dhakal, S., Bigio, A., Blanco, H., Delgado, G. C., Dewar, D., Huang, L., Inaba, A., Kansal, A., Lwasa, S., McMahon, J. E., Müller, D. B., Murakami, J., Nagendra, H., & Ramaswami, A. (2014). Human Settlements, Infrastructure and Spatial Planning. In O. Edenhofer, R. Pichs-Madruga, Y. Sokona, E. Farahani, S. Kadner, K. Seyboth, A. Adler, I. Baum, S. Brunner, P. Eickemeier, B. Kriemann, J. Savolainen, S. Schlömer, C. von Stechow, T. Zwickel, & J.C. Minx (Eds.), *Climate Change 2014: Mitigation of Climate Change. Contribution of Working Group III to the Fifth Assessment Report of the Intergovernmental Panel on Climate Change.* Cambridge University Press, Cambridge, United Kingdom and New York, NY, USA.

Shakti, B. S. (2017). Tackling Violence Against Women: A Study of State Intervention Measures. A comparative study of impact of new laws, crime rate and reporting rate, Change in awareness level. https://wcd.nic.in/sites/default/files/Final%20Draft%20report%20BSS_0.pdf

Stechow, C., Zwickel, T., & Minx, J. C. (Eds.). (2014). Climate Change 2014: Mitigation of Climate Change. Contribution of Working Group III to the Fifth Assessment Report of the Intergovernmental Panel on Climate Change. Cambridge University Press, Cambridge, United Kingdom and New York, NY, USA. https://www.ipcc.ch/site/assets/uploads/2018/02/ipcc_wg3_ar5_chapter12.pdf

Servaes, J. (Ed.). (2017). *Sustainable Development Goals in the Asian Context.* Springer Singapore. https://doi.org/10.1007/978-981-10-2815-1

Singh, K., Bloom, S., & Brodish, P. (2015). Gender equality as a means to improve maternal and child health in Africa. *Health care for women international*, *36*(1), 57–69. https://doi.org/10.1080/07399332.2013.824971

Sinha, A., Sengupta, T., & Alvarado, R. (2020). Interplay between technological innovation and environmental quality: Formulating the SDG policies for next 11 economies. *Journal of Cleaner Production*, *242*, 118549.

Slutskiy, P. (2020). Freedom of Expression, Social Media Censorship, and Property Rights. *Tripodos*, *48*, 53-67. http://doi.org/10.51698/tripodos.2020.48p53-67

Smas, L., Fredricsson, C., & Claessen, H. (2013). Demographic changes, housing policies and urban planning Examples of situations and strategies in Nordic municipalities. NORDREGIO WORKING PAPER 2013, 4. https://www.diva-portal.org/smash/get/diva2:700286/FULLTEXT01.pdf

Smiciklas, J. Menon, M., Carriero, D., Wakhlu, V., Geray, O., Vartto, H., Stankus, A., Galal, H., & Demaithan, H. B. (2017). Connecting cities and communities with the Sustainable Development Goals. CBD, ECLAC, FAO, ITU, UNDP, UNECA, UNECE, UNESCO, UN Environment, UNEP-FI, UNFCCC, UN-Habitat, UNIDO, UNU-EGOV, and WMO. Switzerland, Geneva. https://unece.org/DAM/hlm/documents/Publications/U4SSC_Deliverable-Connecting-Cities-and-Communities.pdf

Spiteri, J. (2020). Early childhood education for sustainability. In W. Leal Filho, A. M. Azul, L. Brandli, P. G. Ozuyar, & T. Wall (Eds.), *Quality Education. Encyclopedia of the UN Sustainable Development Goals*. Switzerland, Cham: Springer

Spiteri, J. (2018). Why we should start early with ESD for lifelong learning. In W. Leal Filho, M. Mifsud, & P. Pace (Eds.), *Handbook of Lifelong Learning for Sustainable Development.* Springer: World Sustainability Series

Sterling, S. (2016). A Commentary on Education and Sustainable Development Goals. *Journal of Education for Sustainable Development*, *10*(2), 208-213. https://doi.org/10.1177/0973408216661886

Stromquist, N. P. (2020). Girls and women in the educational system: The curriculuar challenge. *Prospects*, *49*, 47–50. https://doi.org/10.1007/s11125-020-09482-1

Teotia, M. K. (2015). Housing for the Urban Poor in Chandigarh. *Shelter*, *16*(2), 56-63.

The Economist Intelligence Unit (2019). The critical role of Infrastructure for the Sustainable Development Goals. https://1lib.in/book/5418287/4ece50

Tully, C. (2015). The critical role of effective, accountable and inclusive institutions in implementing the Sustainable Development Goals. Foundation for Democracy and Sustainable Development, London http://www.fdsd.org/wp-content/uploads/2015/10/The-critical-role-of-effective-accountable-and-inclusive-institutions.pdf

UNCTAD, & UNODC (2021). Promotion of international cooperation to combat illicit financial flows and strengthen good practices on asset return to foster sustainable development: Achievement, challenges and way forward. https://unctad.org/system/files/official-document/webosg2021d1_en.pdf

UNESCO (2018a). Situation analysis of SDG4 with a gender lens, Target 4.7. https://unesdoc.unesco.org/ark:/48223/pf0000371227

UNESCO (2018b). World trends in freedom of expression and media development: global report https://unesdoc.unesco.org/ark:/48223/pf0000261065

UNICEF (2009) .A Study on Violence Against Girls Report on the International Girl Child Conference. https://www.unicef-irc.org/publications/pdf/violence_girls_eng.pdf

UNICEF and The Regional Office for CEE/CIS (2015). Children's Equitable Access to Justice: Central and Eastern Europe and Central Asia. UNICEF, Geneva, https://www.unicef.org/media/50996/file/Equitable_access_to_justice_for_children_in_Central_and_Eastern_Europe_and_Central_Asia_-_v2_1.pdf

UNICEF Office of Research (2017). Building the Future: Children and the Sustainable Development Goals in Rich Countries. Innocenti Report Card 14, UNICEF Office of Research – Innocenti, Florence. https://www.unicef-irc.org/publications/pdf/RC14_eng.pdf

United Nations (2018). Working Together: Integration, institutions and the Sustainable Development Goals, World Public Sector Report 2018, Division for Public Administration and Development Management, Department of Economic and Social Affairs, (DPADM), New York. https://publicadministration.un.org/publications/content/PDFs/World%20Public%20Sector%20Report2018.pdf

United Nations (2015). Eliminating corruption is crucial to sustainable development. Press release. United Nations Office on Drugs and Crime. https://www.unodc.org/unodc/en/press/releases/2015/November/eliminating-corruption-is-crucial-to-sustainable-development.html

United Nations (2008). An Introduction to Human Trafficking: Vulnerability, Impact and Action. https://www.unodc.org/documents/human-trafficking/An_Introduction_to_Human_Trafficking_-_Background_Paper.pdf

United Nations Environment Programme (2017). Resilience and Resource Efficiency in Cities. https://wedocs.unep.org/bitstream/handle/20.500.11822/20629/Resilience_resource_efficiency_cities.pdf?sequence=1&isAllowed=y

United Nations Human Rights (2020). Appeal 2021. https://www.ohchr.org/Documents/Publications/AnnualAppeal2021.pdf

United Nations Statistics Division (2016). Goal 11 Make cities and human settlements inclusive, safe, resilient and sustainable. https://unstats.un.org/sdgs/files/metadata-compilation/Metadata-Goal-11.pdf

UN Sustainable Development Goals (2020). *Gender equality grows as key aspect of sustainable ocean management.* https://www.un.org/sustainabledevelopment/blog/2020/03/gender-equality-grows-as-key-aspect-of-sustainable-ocean-management/

United Nations System Chief Executives Board for Coordination (2017). Leaving No One Behind: Equality and Non-Discrimination at the Heart of Sustainable Development: A Shared United Nations System Framework for Action. New York: United Nations.

UN Women (2018a). *Gender Equality and the Sustainable Development Goals in Asia and the Pacific. Baseline and pathways for transformative change by 2030.* https://asiapacific.unwomen.org/en/digital-library/publications/2018/10/apsdg

UN Women (2018b), Turning promises into action: Gender equality in the 2030 Agenda https://www.unwomen.org/en/news/in-focus/women-and-the-sdgs/sdg-14-life-below-water

UN Women (2020a). SDG12: Ensure sustainable consumption and production patterns. https://eca.unwomen.org/en/news/in-focus/women-and-the-sdgs/sdg-12-responsible-consumption-production

UN Women (2020b). *Women and sustainable development goals*. https://sustainabledevelopment.un.org/content/documents/2322UN%20Women%20Analysis%20on%20Women%20and%20SDGs.pdf

Vandenabeele, C., & Lao, C. V. (2007). Legal Identity for Inclusive Development. Asian Development Bank. https://think-asia.org/bitstream/handle/11540/227/legal-identity.pdf?sequence=1

van der Straaten, J. (2015). Legal Identity for All by 2030: How Will We Know? workshop of the Open Society Justice Initiative (OSJI) with support from Civil Registration Centre for Development (CRC4D).

V-Dem Institure (2017). Measuring Corruption in Sustainable Development, Target 16.5 with V-Dem Data. Policy Brief No. #13. https://www.v-dem.net/media/filer_public/a3/1b/a31b2dda-4b98-47fb-b1f4-58f8075e1da7/v-dem_policybrief_13_2017.pdf

V-Dem Institute (2015). Measuring Accountability in Sustainable Development Target 16.6 with V-Dem Data. Policy Brief No. 1. https://www.v-dem.net/media/filer_public/86/99/86995278-8d1a-43df-ae63-3ed723e371bf/sdg_166_policy_brief.pdf

Wang, X., Ren, H., Wang, P., Yang, R., Luo, L., & Cheng, F. (2018). A Preliminary Study on Target 11.4 for UN Sustainable Development Goals. *International Journal of Geoheritage and Parks, 6*(2), 18-24. https://doi.org/10.17149/ijgp.j.issn.2577.4441.2018.02.002

Weiss, T. G., & Wilkinso, R. (2018). The Globally Governed— Everyday Global Governance. *Global Governance*, *24*(2), 193–210. https://doi.org/10.1163/19426720-02402003

Whaites, A. (2016). Achieving the impossible: can we be SDG 16 believers? GovNet Background Paper No2, 2016. OECD. http://www.oecd.org/dac/accountable-effective-institutions/Achieving%20the%20Impossible%20can%20we%20be%20SDG16%20believers.pdf

Woetzel, J., Ram, S., Mischke, J., Garemo, N., & Sankhe, S. (2014). *A blueprint for addressing the global affordable housing challenge*. McKinsey Global Institute. https://www.mckinsey.com/~/media/McKinsey/Featured%20Insights/Urbanization/Tackling%20the%20worlds%20affordable%20housing%20challenge/MGI_Affordable_housing_Full%20Report_October%202014.pdf

World Health Organization (2020). *Global status report on preventing violence against children 2020*. https://resourcecentre.savethechildren.net/node/17789/pdf/9789240004191-eng.pdf

World Health Organization (2011). *World report on disability 2011*. https://disabilityinclusion.msf.org/assets/files/WorldReport_eng.pdf

Zamfir, I. (2020). Peace, justice and strong institutions EU support for implementing SDG 16 worldwide. European Parliamentary Research Service. https://www.europarl.europa.eu/RegData/etudes/BRIE/2020/646156/EPRS_BRI(2020)646156_EN.pdf
